# ﻿Integrative taxonomic revision of the genera *Nesticella* and *Howaia* in Japan with the description of five new species (Araneae, Nesticidae, Nesticellini)

**DOI:** 10.3897/zookeys.1174.101251

**Published:** 2023-08-11

**Authors:** Francesco Ballarin, Katsuyuki Eguchi

**Affiliations:** 1 Systematic Zoology Laboratory, Department of Biological Sciences, Tokyo Metropolitan University, 1-1 Minami-Osawa, Hachioji-shi, 192-0397, Tokyo, Japan Tokyo Metropolitan University Tokyo Japan; 2 Department of Zoology, Museo di Storia Naturale of Verona, Lungadige Porta Vittoria, 9, I-37129 Verona, Italy Museo di Storia Naturale of Verona Verona Italy; 3 Department of International Health and Medical Anthropology, Institute of Tropical Medicine, Nagasaki University, 1-12-4 Sakamoto, Nagasaki-shi, 852-8523, Nagasaki, Japan Nagasaki University Nagasaki City Japan

**Keywords:** COI, phylogeny, Ryukyus, species delimitation, subterranean environment, troglobiont

## Abstract

The Japanese species of the genera *Nesticella* Lehtinen & Saaristo, 1980 and *Howaia* Lehtinen & Saaristo, 1980 (Araneae, Nesticidae, Nesticellini) are revised using an integrative taxonomic approach. Their morphology, phylogenetic position within the genera, assignment to species groups, and distribution in mainland Japan and the Ryukyu islands are discussed herein. A phylogenetic and species delimitation analysis was conducted to confirm the boundaries between the putative species that were initially discriminated based on external and genital morphology. As a result of the present study, six species of *Nesticella* and three species of *Howaia* are proposed for the fauna of Japan based on the combined results of morphology and molecular analyses. Male and female of the previously known species *H.mogera* (Yaginuma, 1972), *N.brevipes* (Yaginuma, 1970), and *N.okinawaensis* (Yaginuma, 1979) are redescribed and illustrated using type specimens or specimens collected at the type locality. *Nesticellaterrestris* (Yaginuma, 1970) is resurrected as a valid species and distinguished from the closely related *N.brevipes* based on morphological and molecular evidence. The male of *N.terrestris* is described for the first time. We herein describe three new species of *Nesticella* and two new species of *Howaia* from different islands in the Ryukyu Archipelago, southwest Japan: *N.insulana***sp. nov.** (♂, ♀) from Yonaguni-jima Island, *N.occulta***sp. nov.** (♀) from Ishigaki-jima Island, *N.silvicola***sp. nov.** (♂, ♀) from Yakushima Island, *H.alba***sp. nov.** (♂, ♀) from Miyako-jima Island and *H.subterranea***sp. nov.** (♀) from Okinoerabu-jima Island. *Nesticellaocculta***sp. nov.**, *H.alba***sp. nov.** and *H.subterranea***sp. nov.** dwell exclusively in caves and show extensive morphological adaptation to subterranean life.

## ﻿Introduction

Nesticidae Simon, 1894 is a relatively small family of scaffold-web spiders currently including 282 species assigned to 16 genera with a nearly worldwide distribution (World Spider Catalog 2023). These spiders often show troglophilic preferences and are frequently associated with caves or other dark and humid habitats. The taxonomy and phylogeny of this family has received increasing interest in the last decade by international arachnologists resulting in the publication of numerous new studies (e.g., Liu and Li 2013; Ballarin and Li 2015, 2018; Grall and Jäger 2016; Lin et al. 2016; Pavlek and Ribera 2017; Ribera 2018; Ballarin 2020; Suzuki and Ballarin 2020; Esyunin and Efimik 2022; Fomichev et al. 2022; Ramírez et al. 2022; Zigler and Milne 2022; Hedin and Milne 2023; Ribera and Dimitrov 2023; Sherwood et al. 2023). Such efforts have allowed the description of several new taxa and a progressive revision and redefinition of the family.

The tribe Nesticellini Lehtinen & Saaristo, 1980, also known as “short-legged” nesticids, currently includes two main genera: *Nesticella* Lehtinen & Saaristo, 1980 and *Howaia* Lehtinen & Saaristo, 1980. *Nesticella* is the second largest genus among Nesticidae (World Spider Catalog 2023) and it shows considerable heterogeneity in the shape of male palp and female epigyne. Currently *Nesticella* involves 66 valid species provisionally grouped into four species groups (sensu Lin et al. 2016) based on the morphology of the genitalia. Such diversity, together with the results of preliminary molecular analysis, suggests that *Nesticella* is potentially paraphyletic and that its classification still needs a comprehensive revision (Lin et al. 2016; Ballarin and Li 2018, see also Lehtinen and Saaristo 1980). In this regard, just recently eight species of the *Nesticellamogera* group (sensu Lin et al. 2016) have been transferred to the revalidated genus *Howaia* by Sherwood et al. (2023). Although also distributed in South America, Africa, Europe (introduced), and Oceania, Nesticellini spiders have the greatest taxonomic and ecologic diversity in Asia, where the vast majority of the known species occur (52 species, ~80% of the total) (Lin et al. 2016; World Spider Catalog 2023). The *Nesticella* and *Howaia* fauna of China and Indochina has been progressively explored in recent years leading to the discovery of several new species and allowing a better understanding of their phylogenetic relationships (Liu and Li 2013; Zhang and Li 2013; Ballarin and Li 2015, 2018; Grall and Jäger 2016; Lin et al. 2016; Wang et al. 2022). However, the species distributed in other regions of the Asian continent and adjacent islands and archipelagos have generally been poorly investigated and often neglected. Among the Asian regions where the nesticid fauna is still poorly known are South Asia and the Malay, Philippines, and the Japanese archipelagos.

Currently, 59 species and subspecies assigned to six genera of Nesticidae have been recorded in mainland Japan, the Ryukyus, and other surrounding islands (Suzuki and Ballarin 2020; Tanikawa 2023; World Spider Catalog 2023). This means that Japan alone hosts more than 20% of the worldwide nesticid species, making it one of the hotspots of diversity for this family. Nevertheless, only two species of *Nesticella* and one of *Howaia* are currently reported on the Japanese islands: *Nesticellabrevipes* (Yaginuma, 1970), *N.okinawaensis* (Yaginuma, 1979), and *H.mogera* (Yaginuma, 1972). *Nesticellabrevipes* and *H.mogera* are broadly recorded across mainland Japan and nearby countries, including China, Korea, and the Russian Far East (Shinkai et al. 2022; World Spider Catalog 2023). In contrast, *N.okinawaensis* is distributed only on Okinawa-honto and Kume-jima islands in the Ryukyus (Tanikawa and Sasaki 1999; Shinkai et al. 2022). These species inhabit moist and shaded places, including caves, mines, screes, forest leaf litter, and, in the case of *H.mogera*, meadows, paddy fields, vegetated seashores, and hedgerows in city parks. Although *Nesticella* and *Howaia* species can be relatively easy to find in their suitable habitats, the latest extensive research on these genera dates back to the 1970s with studies by Yaginuma (1970, 1972, 1979). No taxonomic research dealing with Japanese fauna has been conducted in the last 40 years, and no molecular phylogenetic hypothesis involving the Japanese species is currently available.

In the course of our surveys on spiders dwelling in forest litter and caves in Japan, we had the opportunity to collect several *Nesticella* and *Howaia* specimens from different localities and islands. Among them, we recognized morphologically unique forms likely representing undescribed species. Here, we revise the Japanese *Nesticella* and *Howaia* based on the type specimens of the species described in Japan and the newly collected material. The goals of our work are as follows: (i) confirm the boundaries of *Nesticella* and *Howaia* species living in Japan and assign them to the known species groups; in doing so we use an integrative taxonomic approach consisting of conventional morphological examination and COI-based DNA barcoding, (ii) redescribe and illustrate the Japanese named species, using a more modern approach, and (iii) describe and name the species recognized as new by the integrative taxonomic analyses. In addition, we provide supplementary information about the ecology, habitat, and other biological characteristics of the species herein discussed. Such information is often lacking in taxonomic revisions but may represent an important additional source of data for studies on the systematic and evolutionary history of arthropod taxa.

## ﻿Materials and methods

### ﻿Taxonomy

Fresh specimens were collected by sieving the forest leaf litter with an entomological litter reducer or, in caves, by hand with visual searching. When possible, environmental variables (e.g., temperature and humidity) were recorded inside the caves using an As One TH-220 Portable thermo-hygrometer. Additional specimens were borrowed from the collections of the National Museum of Nature and Science of Japan and the Osaka Museum of Natural History, or were kindly provided by local researchers. Newly collected specimens were preserved in 99% ethanol and stored in freezers at -20 °C in the laboratory of Systematic Zoology, Department of Biological Sciences, Tokyo Metropolitan University, Japan (**TMU**) for molecular and morphological analyses. Individuals were examined using a Nikon SMZ1270 and an AZ100 stereo microscopes at the same institute. Epigynes were dissected using a sharp needle and cleared by boiling them for a few minutes in a 20% KOH solution until the inner structures were visible. Epigynes and vulvae were subsequently observed using a Nikon Optiphot 2 microscope. Photographs were taken using a Canon EOS kiss X10 digital camera mounted on AZ100 or Optiphot 2 microscopes. Final images were assembled using Helicon Focus v. 7 image stacking software (https://www.heliconsoft.com) and edited with Adobe Photoshop CC v. 20.0.6 (https://www.photoshop.com/). Lengths of leg segments were measured on the lateral side and are given as follows: total length (femur, patella, tibia, metatarsus, tarsus). All measurements in the text are given in millimeters. The size scatterplot was visualized using Microsoft Excel.

All vouchers used in this study are preserved in the following collections: the National Museum of Nature and Science, Tsukuba (**NMST**), the Museum of Nature and Human Activities, Hyogo (**MNHAH**), the Ryukyu University Museum Fujukan, Okinawa (**RMUF**), the Osaka Museum of Natural History, Osaka (**OMNH**), and in the personal collections of Francesco Ballarin (**FBPC**), Yuya Suzuki (**YSPC**), Toshimichi Nagai (**TNPC**), and Yuri M. Marusik (**YMPC**). The nomenclature of morphological characters used in this work follows those of Suzuki and Ballarin (2020) and Fomichev et al. (2022). The abbreviations used in the text and figures are as follows:

**ALE** anterior lateral eyes;

**AME** anterior median eyes;

**BV** maximum likelihood bootstrap value;

**Ca** apophysis retrolateral process of conductor;

**Cd** copulatory duct;

**Cl** lobe of conductor;

**Cm** median process of conductor;

**Co** copulatory opening;

**Cp** prolateral process of conductor;

**Cr** retrolateral process of conductor;

**Di (I–II)** distal process(es) of paracymbium;

**Do** dorsal process of paracymbium;

**E** embolus;

**Id** insemination duct;

**P** paracymbium;

**PLE** posterior lateral eyes;

**PME** posterior median eyes;

**PP** Bayesian Inference posterior probability;

**Ra** radical apophysis;

**Rx** radix;

**S** spermatheca;

**Sc** scapus;

**Sd** sperm duct;

**St** subtegulum;

**Te** tegulum;

**Ve (I–II)** ventral process(es) of paracymbium.

### ﻿Molecular analysis

Phylogenetic analyses, species delimitation analyses, and a pairwise distance genetic divergence analysis were conducted to confirm the boundaries and intraspecific genetic diversity of the species tentatively discriminated by examining external and genital morphology (hereafter referred to as “morphospecies”). For each species, fresh representative specimens from the type locality or nearby localities (hereafter collectively referred to as “topotypes”) were also included in the analyses whenever possible. The species *Howaiasubterranea* sp. nov. was excluded from the analyses because of the lack of fresh samples. Total genomic DNA was extracted from the leg tissue using a Chelex-TE-ProK method. Four legs were removed from each specimen and included in an extraction buffer of 100 µL containing a solution of 10% Chelex-TE and 4 µL Qiagen Proteinase K. The extraction buffer was incubated at 56 °C for 24 h followed by 10 min at 99 °C to inactivate the Proteinase K. A fragment of the mitochondrial gene Cytochrome c oxidase subunit I (COI, 1200 bp) was selectively amplified using the following primer couples: LCO1490 (F) GGTCAACAAATCATCATAAAGATATTGG (Folmer et al. 1994) and CHR2 (R) GGATGGCCAAAAAATCAAAATAAATG (Barrett and Hebert 2005), C1-J-2183 (F) CAACATTTATTTTGATTTTTTGG (Simon et al. 1994), and C1-N-2776 (R) GGATAATCAGAATATCGTCGAGG (Hedin and Maddison 2001).

A SimpliAmp Thermal Cycler (Thermo Fisher Scientific, U.S.) with a final volume of 11 µl was used for the PCR amplification under the following protocol: 94° (2’); [98° (10’’), 45° (30’’), 68° (10’’)] ×5; [94° (10’’), 50° (30’’), 68° (45’’)] ×40; 68° (7’). Cycle sequencing reactions were performed using a “SupreDye Cycle Sequencing Kit.” All experiments were carried out in the Systematic Zoology and Systematic Botany Laboratories of TMU, Japan. Additional sequences were obtained from previous phylogenetic studies on Asian *Nesticella* species (Zhang and Li 2013; Ballarin and Li 2018). The complete list of the specimens used is reported in Table 1. Sequences were visually checked and aligned using the online version of MAFFT software v. 7 (https://mafft.cbrc.jp/alignment/server/) under the G-INS-I method.

**Table 1.** list of specimens used in this study and related GenBank accession numbers and locality of collection. New sequences are indicated by an asterisk.

code	Species	GenBank	Locality	Origin
001	*Howaiayanbeiensis*	MG200877	China, Guangxi Prov., Yanbei cave	Ballarin and Li 2018
004	*Nesticellaapiculata*	MG200892	China, Beijing Municipality, cave without name	Ballarin and Li 2018
007	*Nesticellabeccus*	MG200985	Laos, Khammouanc Prov., Tham Kamouk cave	Ballarin and Li 2018
009	*Nesticellabeccus*	MG200986	Laos, Bolikhamxay Prov., Hospital cave	Ballarin and Li 2018
020	*Nesticellakaohsiungensis*	MG200979	Taiwan, Nantou County, Huisunlinchang forest	Ballarin and Li 2018
023	*Nesticellaapiculata*	MG200894	China, Jiangxi Prov., Longgong cave	Ballarin and Li 2018
034	*Nesticellasongi*	MG200912	China, Guangxi Prov., Feng cave	Ballarin and Li 2018
070	*Nesticellabeccus*	MG200987	China, Yunnan Prov., Xishuangbanna Nature Reserve	Ballarin and Li 2018
077	*Nesticellabeccus*	MG200988	China, Yunnan Prov., Xishuangbanna Nature Reserve	Ballarin and Li 2018
083	*Nesticellaapiculata*	MG200895	China, Jiangxi Prov., Yuhu cave	Ballarin and Li 2018
091	*Nesticellaodonta*	MG200934	China, Jiangxi Prov., Xiongxin cave	Ballarin and Li 2018
092	*Nesticellaodonta*	MG200937	China, Jiangxi Prov., cave without name	Ballarin and Li 2018
095	*Nesticellaverticalis*	MG200922	China, Guizhou Prov., Dongfushanzhuang cave	Ballarin and Li 2018
113	*Nesticellashanlinensis*	MG200962	China, Guizhou Prov., Menglong cave	Ballarin and Li 2018
121	*Nesticellaconnectens*	MG201016	Thailand, Satun Prov., Beating cave	Ballarin and Li 2018
135	*Nesticellashanlinensis*	MG200963	China, Chongqing Municipality, Erlong cave	Ballarin and Li 2018
197	*Nesticellaaelleni*	MG201003	Sri Lanka, Sabaragamuwa Prov., Isthripura cave	Ballarin and Li 2018
198	*Nesticellaaelleni*	MG201004	Sri Lanka, Central Prov., Peraderiya Town, Botanic Gardens	Ballarin and Li 2018
199	*Nesticellaaelleni*	MG201007	Sri Lanka, Southern Prov., Yabbalamulla	Ballarin and Li 2018
200	*Nesticellaaelleni*	MG201006	Sri Lanka, Western Prov., Avissawella	Ballarin and Li 2018
208	*Nesticellakaohsiungensis*	MG200981	Taiwan, Kaohsiung City, cave without name	Ballarin and Li 2018
209	*Nesticellakaohsiungensis*	MG200980	Taiwan, Nantou County, Huisun forest area	Ballarin and Li 2018
282	*Nesticellasongi*	MG200913	China, Guizhou Prov., cave without name	Ballarin and Li 2018
291	*Nesticellashanlinensis*	MG200964	China, Guizhou Prov., Woshuida cave	Ballarin and Li 2018
400	*Nesticellaaelleni*	MG201005	Sri Lanka, Central Prov., Koththol cave	Ballarin and Li 2018
412	*Nesticellaapiculata*	MG200893	China, Henan Prov., Yin cave	Ballarin and Li 2018
414	*Howaiahuomachongensis*	MG200881	China, Hubei Prov., cave without name	Ballarin and Li 2018
417	*Howaiahuomachongensis*	MG200884	China, Hubei Prov., Xiejiaba Village, cave without name	Ballarin and Li 2018
419	*Howaiahuomachongensis*	MG200883	China, Hubei Prov., Guanyin cave	Ballarin and Li 2018
420	*Nesticellaconnectens*	MG201020	Thailand, Phang Nga Prov., Tharn Lod cave	Ballarin and Li 2018
421	*Nesticellaconnectens*	MG201021	Thailand, Krabi Prov., Blue Diamond cave	Ballarin and Li 2018
422	*Nesticellaconnectens*	MG201015	Thailand, Nakhon Nayon Prov., Changwat Sakhon Nayok forest	Ballarin and Li 2018
439	*Howaiahuomachongensis*	MG200882	China, Hubei Prov., Xianwu cave	Ballarin and Li 2018
453	*Nesticellashanlinensis*	MG200966	China, Chongqing Municipality, Jin’e cave	Ballarin and Li 2018
454	*Howaiamogera*	MG200905	China, Sichuan Prov., Baxian cave	Ballarin and Li 2018
479	*Nesticellabeccus*	MG200989	China, Yunnan Prov., Laohu cave	Ballarin and Li 2018
489	*Nesticellabeccus*	MG200990	China, Yunnan Prov., Niumo cave	Ballarin and Li 2018
507	*Nesticellaodonta*	MG200936	China, Yunnan Prov., Manglehe cave	Ballarin and Li 2018
528	*Nesticellaodonta*	MG200939	China, Sichuan Prov., Shihai Township	Ballarin and Li 2018
563	*Nesticellaverticalis*	MG200925	China, Guizhou Prov., Guyang cave	Ballarin and Li 2018
572	*Nesticellaconnectens*	MG201017	Thailand, Yala Prov., outside the Khao Thai cave	Ballarin and Li 2018
573	*Nesticellaconnectens*	MG201018	Thailand, Yala Prov., outside the Krasaeng cave	Ballarin and Li 2018
574	*Nesticellaconnectens*	MG201019	Thailand, Rattalung Prov., Lor Kor cave	Ballarin and Li 2018
1414	*Howaiamogera*	MG200897	China, Hubei Prov., cave without name	Ballarin and Li 2018
BL3	*Nesticellashanlinensis*	MG200967	China, Guizhou Prov., Guihua Village, cave without name	Ballarin and Li 2018
BS	*Nesticellasongi*	MG200914	China, Guizhou Prov., Shuibashui cave	Ballarin and Li 2018
GX7	*Howaiamogera*	MG200898	China, Guizhou Prov., Tiexice cave	Ballarin and Li 2018
HE	*Nesticellaverticalis*	MG200923	China, Guizhou Prov., Hei cave	Ballarin and Li 2018
HM	*Nesticellaverticalis*	MG200927	China, Hunan Prov., Hama cave	Ballarin and Li 2018
HU	*Howaiahuomachongensis*	MG200885	China, Hunan Prov., Huanglong cave	Ballarin and Li 2018
LI	*Nesticellaodonta*	MG200938	China, Guizhou Prov., Liuguan cave	Ballarin and Li 2018
ND	*Nesticellaverticalis*	MG200926	China, Guizhou Prov., Niu cave	Ballarin and Li 2018
Ne01	*Nesticellabrevipes*	OR123601*	Japan, Kyushu, Ōita Pref., Odzu Tome dōkutsu iseki cave	This work
Ne02	*Nesticellabrevipes*	OR123602*	Japan, Kyushu, Ōita Pref., Odzu Tome dōkutsu iseki cave	This work
Ne10	*Nesticellaterrestris*	OR123603*	Japan, Tokyo, Hachioji	This work
Ne11	*Howaiamogera*	OR123604*	Japan, Shizuoka Pref., Okuyama	This work
Ne12	*Howaiamogera*	OR123605*	Japan, Fukui Pref., Nagatani	This work
Ne13	*Howaiamogera*	OR123606*	Japan, Mie Pref., Otani	This work
Ne16	*Howaiamogera*	OR123607*	Japan, Yamagata Pref., Oyama	This work
Ne17	*Howaiamogera*	OR123608*	Japan, Nagano Pref., Ijima	This work
Ne18	*Nesticellaterrestris*	OR123609*	Japan, Saitama Pref., Furutera cave	This work
Ne26	*Howaiamogera*	OR123610*	Japan, Nagano Pref., Ijima	This work
Ne27	*Howaiamogera*	OR123611*	Japan, Nagano Pref., Ijima	This work
Ne39	*Nesticellaokinawaensis*	OR123612*	Japan, Amami Is., Naze	This work
Ne40	*Howaiaalba*	OR123613*	Japan, Miyakojima Is., Tsuzupisuki-abi cave	This work
Ne41	*Nesticellaocculta*	OR123614*	Japan, Ishigaki Is., Kabirano-ana cave	This work
Ne42	*Howaiamogera*	OR123615*	Japan, Ishigaki Is., Fukubukuīzā cave	This work
Ne43	*Howaiamogera*	OR123616*	Japan, Ishigaki Is., Fukubukuīzā cave	This work
Ne44	*Nesticellaocculta*	OR123617*	Japan, Ishigaki Is., Kabirano-ana cave	This work
Ne45	*Nesticellaokinawaensis*	OR123618*	Japan, Okinawa-honto Is., Izumi	This work
Ne46	*Nesticellaokinawaensis*	OR123619*	Japan, Okinawa-honto Is., Izumi	This work
Ne47	*Howaiaalba*	OR123620*	Japan, Miyakojima Is., Pinza-abu cave	This work
Ne47b	*Howaiaalba*	OR123621*	Japan, Miyakojima Is., Pinza-abu cave	This work
Ne48	*Howaiaalba*	OR123622*	Japan, Miyakojima Is., Tsuzupisuki-abu cave	This work
Ne49	*Nesticellaokinawaensis*	OR123623*	Japan, Okinawa-honto Is., Yona, Yambaru Park	This work
Ne50	*Nesticellaokinawaensis*	OR123624*	Japan, Okinawa-honto Is., Yona, Yambaru Park	This work
Ne51	*Howaiamogera*	OR123625*	Japan, Yonaguni Is., Irizaki	This work
Ne52	*Nesticellainsulana*	OR123626*	Japan, Yonaguni Is., Mitsudahara Forest Park	This work
Ne53	*Nesticellainsulana*	OR123627*	Japan, Yonaguni Is., Mitsudahara Forest Park	This work
Ne54	*Nesticellabrevipes*	OR123628*	Japan, Shikoku Is., Konji	This work
Ne55	*Howaiamogera*	OR123629*	Japan, Okinawa-honto Is., Naha, Sueyoshi park	This work
Ne56	*Howaiamogera*	OR123630*	Japan, Hachijo-jima Is., Hachijo Botanical Park	This work
Ne59	*Nesticellaterrestris*	OR123631*	Japan, Aichi Pref., Suse-cho	This work
Ne60	*Nesticellaterrestris*	OR123632*	Japan, Tokyo Pref., Hachioji, Hachioji castle ruins	This work
Ne61	*Nesticellaterrestris*	OR123633*	Japan, Tokyo Pref., Hachioji, Hachioji castle ruins	This work
Ne62	*Howaiamogera*	OR123634*	Japan, Amami-Oshima Is., Kasarichō Ōaza Kise	This work
Ne65	*Nesticellasilvicola*	OR123635*	Japan, Yakushima Is., Koseda	This work
Ne66	*Nesticellasilvicola*	OR123636*	Japan, Yakushima Is., Anbo	This work
Ne67	*Nesticellasilvicola*	OR123637*	Japan, Yakushima Is., Isso	This work
Ne68	*Nesticellasilvicola*	OR123638*	Japan, Yakushima Is., Miyanoura	This work
Ne69	*Howaiamogera*	OR123639*	Japan, Yakushima Is., Anbo	This work
Ne70	*Nesticellaokinawaensis*	OR123640*	Japan, Okinawa-honto Is.,Yona, Yambaru Park	This work
Ne71	*Nesticellaokinawaensis*	OR123641*	Japan, Okinawa-honto Is.,Yona, Yambaru Park	This work
Ne72	*Nesticellaokinawaensis*	OR123642*	Japan, Okinawa-honto Is.,Yona, Yambaru Park	This work
Ne77	*Howaiaalba*	OR123643*	Japan, Miyako-jima Is., Nakabari Limestone cave	This work
Ne78	*Howaiaalba*	OR123644*	Japan, Miyako-jima Is., Tsuzupisuki-abu cave	This work
Ne79	*Howaiamogera*	OR123645*	Japan, Okinawa-honto Is. Shimuku Gama cave	This work
Ne81	*Nesticellaokinawaensis*	OR123646*	Japan, Kumejima Is., Maja	This work
Ne82	*Nesticellaokinawaensis*	OR123647*	Japan, Kumejima Is., Uegusuku	This work
Neque	*Nesticellaquelpartensis*	JN817085	South Korea, Jeju Island, cave	GenBank
QX1	*Howaiayanbeiensis*	MG200880	China, Guangxi Prov., Qixing cave	Ballarin and Li 2018
QX2	*Howaiayanbeiensis*	MG200878	China, Guangxi Prov., Dushu cave	Ballarin and Li 2018
QX4	*Howaiayanbeiensis*	KF359049	China, Guangxi Prov., Ruyan cave	Zhang and Li 2013
SH2	*Nesticellabeccus*	KF359041	China, Yunnan Prov., Xianfo cave	Zhang and Li 2013
SH6	*Nesticellabeccus*	KF359038	China, Yunnan Prov., Riyue cave	Zhang and Li 2013
SH7	*Nesticellabeccus*	KF359039	China, Yunnan Prov., Shihua cave	Zhang and Li 2013
SI	*Nesticellasongi*	MG200915	China, Guizhou Prov., Shuilong Cave	Ballarin and Li 2018
WA	*Nesticellashanlinensis*	MG200965	China, Guizhou Prov., Mawan cave	Ballarin and Li 2018
YF3	*Nesticellasongi*	MG200916	China, Guangxi Prov., Dayan cave	Ballarin and Li 2018
YG	*Nesticellaverticalis*	MG200924	China, Guizhou Prov., Yangzi cave	Ballarin and Li 2018
YN	*Howaiahuomachongensis*	MG200886	China, Hunan Prov., Yanzi cave	Ballarin and Li 2018
YS	*Nesticellaodonta*	MG200935	China, Hunan Prov., Changsha City	Ballarin and Li 2018
Z062	*Wraioslongiembolus*	MG201040	China, Yunnan Prov., Xishuangbanna Nature Reserve	Ballarin and Li 2018

We conducted a phylogenetic analysis using a final dataset of 113 terminals assigned to 20 morphospecies. The nesticid *Wraioslongiembolus* Ballarin & Li, 2015 was used as an outgroup to root the trees because of its close relationship with *Nesticella* and *Howaia* (Ballarin and Li 2018). We inferred two distinct phylogenies using a maximum likelihood (ML) analysis in RAxML-NG (Kozlov et al. 2019) and a Bayesian inference (BI) analysis in MrBayes v. 3.2.7 (Ronquist et al. 2012). Both analyses were run remotely on CIPRES Science Gateway v. 3.3 (https://www.phylo.org/). We used an unpartitioned dataset since preliminary tests suggested no significative differences in the tree topologies and nodes support using a single partition or a partition by codon. ML was performed using a rapid bootstrap of 1,000 replicates under a GTRGAMMAI model and the standard parameters suggested by the software. BI was performed twice with the run of four Monte Carlo Markov chains (MCMCs) for one million generations with a 25% burning fraction and sampling trees every 1,000 generations. We monitored the results using TRACER v. 1.7.1 (Rambaut et al. 2018) confirming that the analysis has reached an effective sample size (ESS) of more than 200 in order to ensure chains convergence. A TIM3+I+G substitution model was used in the analysis as suggested by Jmodeltest2 (Darriba et al. 2012) testing the dataset under the corrected Akaike’s Information Criterion (AICc). The resulting trees were displayed using Figtree software v. 1.4.3 (http://tree.bio.ed.ac.uk/software/ﬁgtree/). Nodes with BV ≥ 70 or PP ≥ 0.95 were considered as highly supported.

The species delimitation analyses were performed comparing three different methods: ASAP, mPTP, and bPTP. These are among the most frequently used methods for single-locus species delimitation in modern studies. The Assemble Species by Automatic Partitioning (ASAP) analysis (Puillandre et al. 2021) was performed online (https://bioinfo.mnhn.fr/abi/public/asap/) under Jukes-Cantor (JC69) and Kimura (K80) models. A trimmed dataset of 650 bp (= the Folmer region) was used to minimize the effects of missing sites at the terminals that remained involved in the original dataset. Split group probability was set to 2% and other options were set as default. Following the ASAP manual, we considered only the output with the lowest score (SC = 4.5).

Both the mPTP and bPTP methods represent improved versions of the older Poisson Tree Processes method (PTP). They use respectively a multi-rate Poisson method (mPTP) (Kapli et al. 2017) and Bayesian support values (bPTP) (Zhang et al. 2013) assuming independent exponential distributions to model the branch lengths of each species. The Newick input tree was reconstructed using MrBayes based on the same trimmed dataset previously used for the ASAP analysis and under the same conditions described for the phylogenetic analysis. We performed the mPTP analysis online (https://mptp.h-its.org/#/tree) using the default parameters and cropping the outgroup. The bPTP analysis was also run remotely (https://species.h-its.org/ptp/). The number of MCMC generations was set to 200,000 with the thinning and the burn-in values set respectively to 100 and 0.1. Other parameters were set to default.

Finally, we performed an uncorrected pairwise-distance genetic divergence analysis in MEGA X v. 10.0.5 (Kumar et al. 2018). We used the same dataset for the species delimitation analysis to calculate intraspecific divergences. We prepared a further reduced dataset obtained by pruning the samples to have a single sequence of the COI barcode for each species to calculate interspecific divergences. The sequences of the “topotypes” were preferentially kept when possible. A bootstrap method with 1,000 replications was conducted with the other options set as default.

## ﻿Results and discussion

### ﻿Remarks on the phylogeny of *Nesticella* and *Howaia*

The phylogenetic trees generated using both RAxML and MrBayes show the exact same topology and a similar high support for the majority of the nodes. The resulting combined tree is reported in Fig. 14. The morphospecies included in this study cluster together into five major lineages, three of which correspond to the species groups sensu Lin et al. (2016) and one to *Howaia* (= *N.mogera* group). These clades are highly supported by at least one of the two phylogenetic inferences. Our results support the monophyly of *Howaia* with the species belonging to the *N.mogera* group all sharing the same recent common ancestor. In accordance with previous studies, *Nesticella* is resolved as paraphyletic being divided into several distinct lineages and with *Howaia* nested inside. Such results further highlight that *Nesticella* is in need of a proper systematic revision. Among the *Nesticella* clades, *N.okinawaensis* is recovered as a well-supported independent lineage (BV = 78, PP = 1), distinct from the *N.brevipes* group. This suggests a modification to the temporary placement of *N.okinawaensis* in the *N.brevipes* group by Lin et al. (2016). The genetic distance between different *Nesticella* species groups and *Howaia* ranges from 14% to 19% with a mean of 16%.

### ﻿Recognition of putative species using an integrative approach

**Figure 1.**
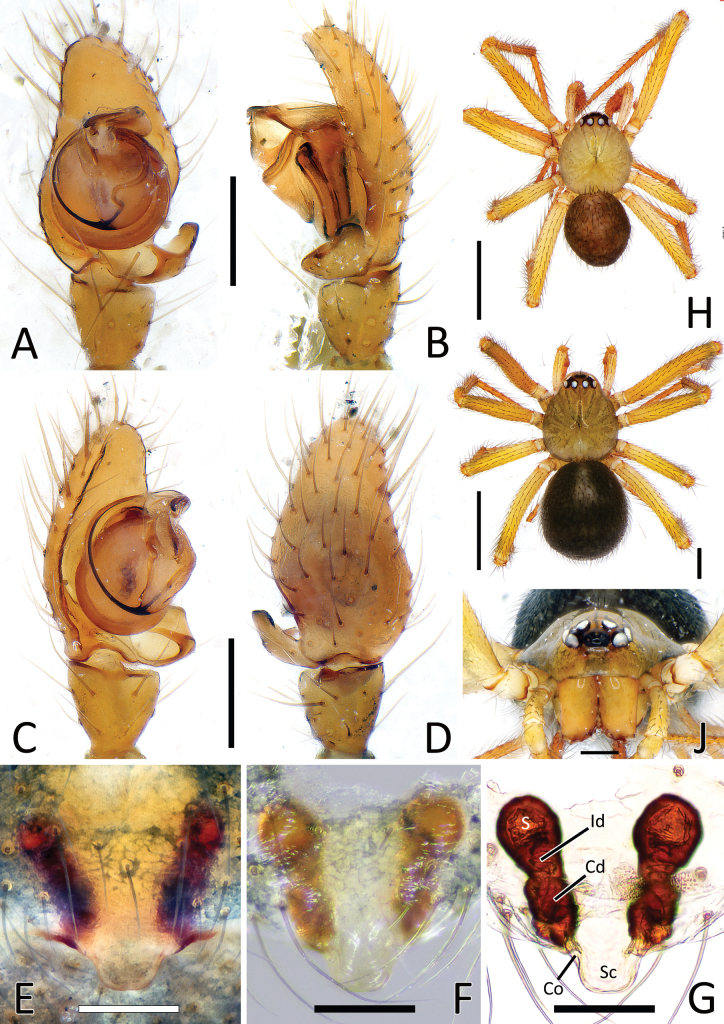
*Howaiamogera***A** male palp (holotype), ventral view **B** same, retrolateral view **C** same, ventro-prolateral view **D** same, dorsal view **E** female epigyne ventral view (specimen from Tokyo area) **F** same after dissection, shape variation **G** vulva, dorsal view **H** habitus of male (specimen from Tokyo area) **I** habitus of female **J** cephalic area of female, frontal view. Abbreviations: Cd – copulatory duct; Co – copulatory opening; Id – insemination duct; S – spermatheca; Sc – scapus. Scale bars: 0.2 mm (**A–G, J**); 1.0 mm (**H, I**).

**Figure 2.**
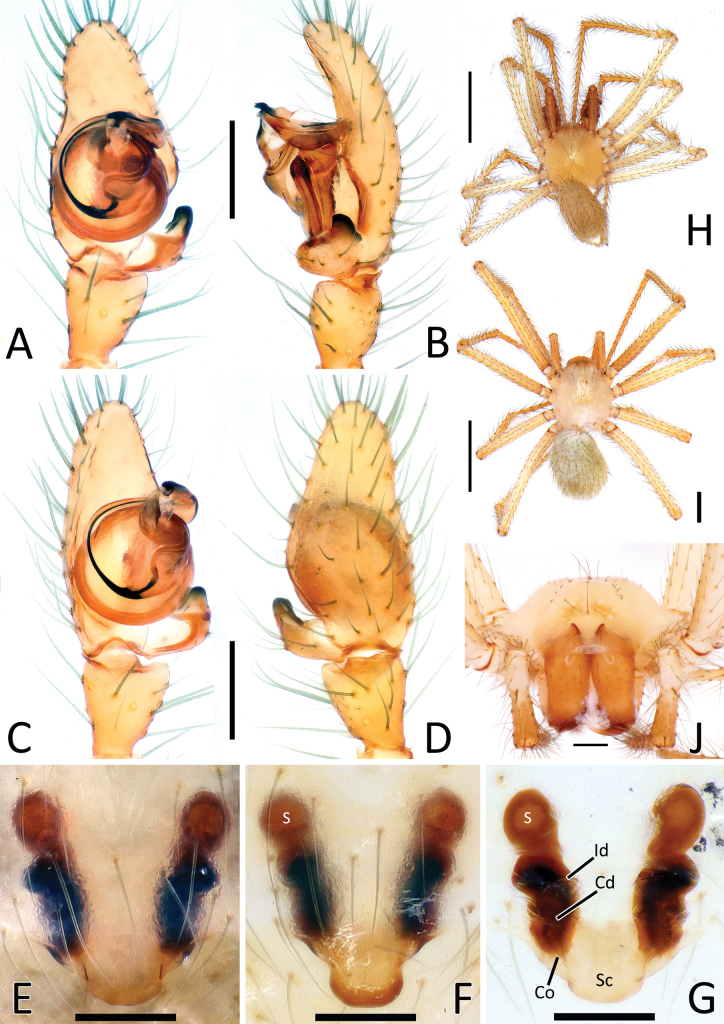
*Howaiaalba* sp. nov. **A** male palp (holotype), ventral view **B** same, retrolateral view **C** same, ventro-prolateral view **D** same, dorsal view **E** female epigyne (one of the paratypes), ventral view **F** same, shape variation **G** vulva, dorsal view **H** habitus of male (holotype) **I** habitus of female (one of the paratypes) **J** cephalic area of female, frontal view. Abbreviations: Cd – copulatory duct; Co – copulatory opening; Id – insemination duct; S – spermatheca; Sc – scapus. Scale bars: 0.2 mm (**A–G, J**); 1.0 mm (**H, I**).

**Figure 3.**
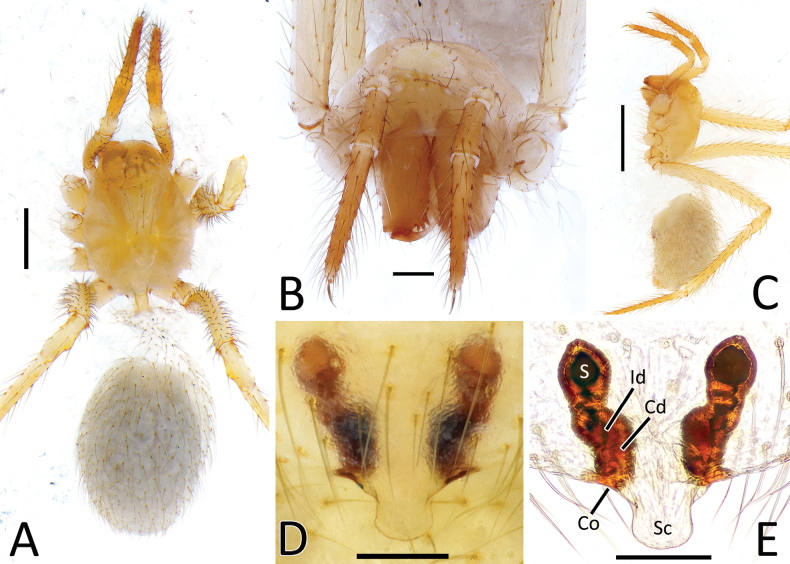
*Howaiasubterranea* sp. nov. **A** habitus of female (holotype) **B** cephalic area of female, frontal view **C** habitus of female, lateral view **D** female epigyne, ventral view **E** vulva, dorsal view. Abbreviations: Cd – copulatory duct; Co – copulatory opening; Id – insemination duct; S – spermatheca; Sc – scapus. Scale bars: 1.0 mm (**A, C**); 0.2 mm (**B, D, E**).

**Figure 4.**
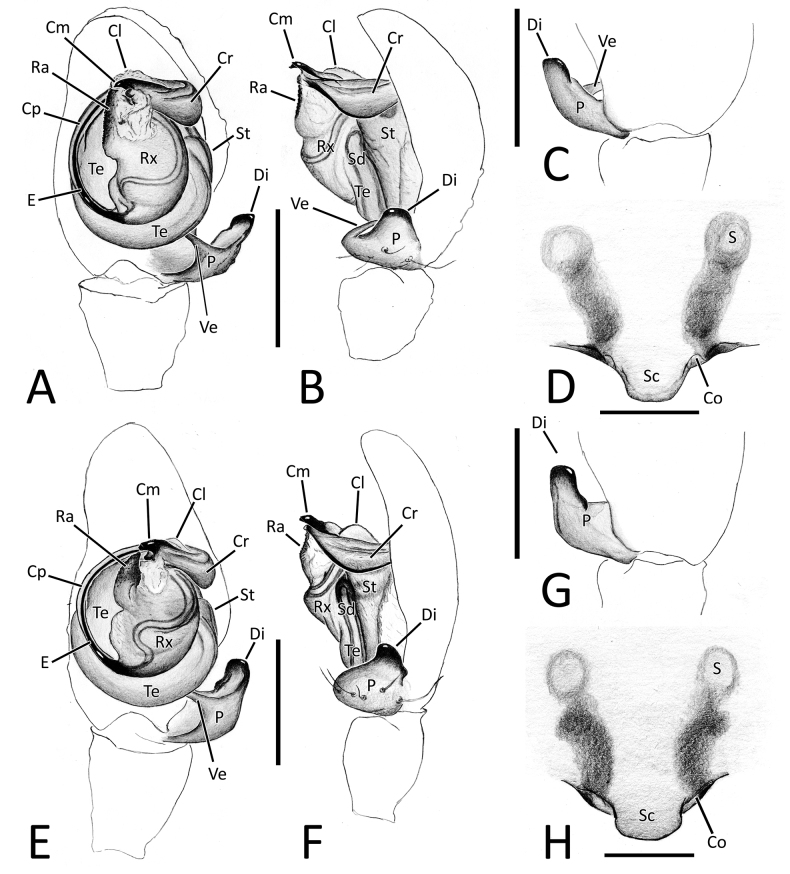
Genitalia of *Howaiamogera* and *H.alba* sp. nov. **A** male palp of *H.mogera*, ventral view **B** same, retrolateral view **C** detail or paracymbium, dorsal view **E** female epigyne, ventral view **E** male palp of *H.alba* sp. nov., ventral view **F** same, retrolateral view **C** detail or paracymbium, dorsal view **H** female epigyne, ventral view. Abbreviations: Cl – lobe of conductor; Cm – median process of conductor; Co – copulatory opening; Cp – prolateral process of conductor; Cr – retrolateral process of conductor; Di – distal process of paracymbium; E – embolus; P – paracymbium; Ra – radical apophysis; Rx – radix; S – spermatheca; Sc – scapus; Sd – sperm duct; St – subtegulum; Te – tegulum; Ve – ventral process of paracymbium. Scale bars: 0.2 mm.

**Figure 5.**
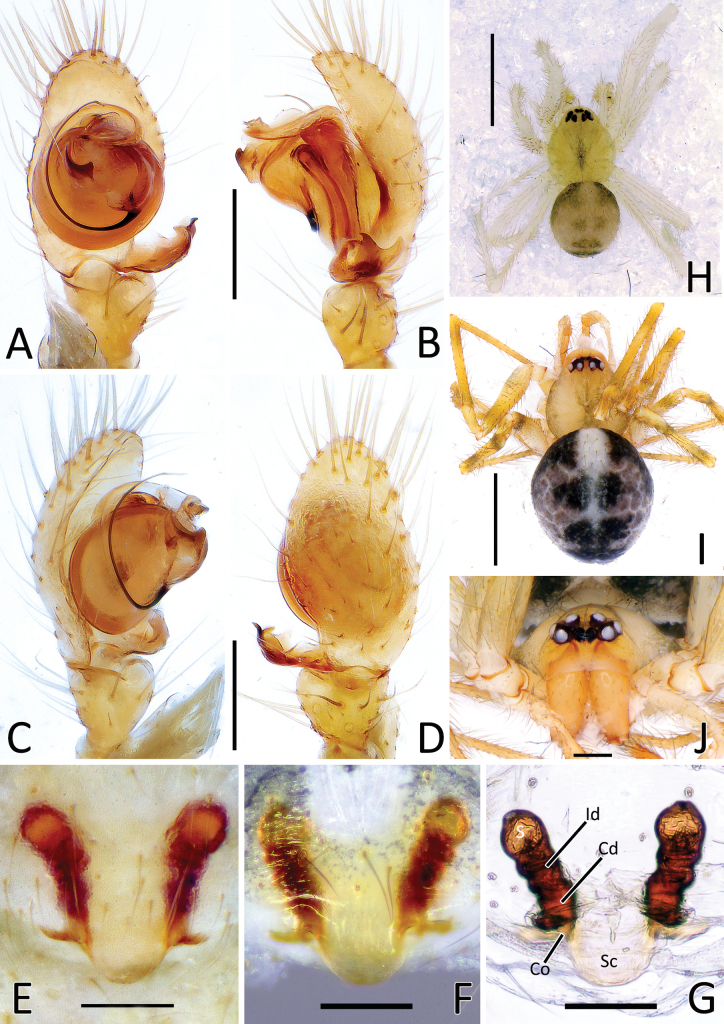
*Nesticellabrevipes***A** male palp (paratype), ventral view **B** same, retrolateral view **C** same, ventro-prolateral view **D** same, dorsal view **E** female epigyne (holotype), ventral view **F** same, female from Kyushu **G** vulva, dorsal view **H** habitus of male (old specimen from Kyushu); I habitus of female (specimen from Shikoku); J cephalic area of female, frontal view. Abbreviations: Cd – copulatory duct; Co – copulatory opening; Id – insemination duct; S – spermatheca; Sc – scapus. Scale bars: 0.2 mm (**A–G, J**); 1.0 mm (**H, I**).

**Figure 6.**
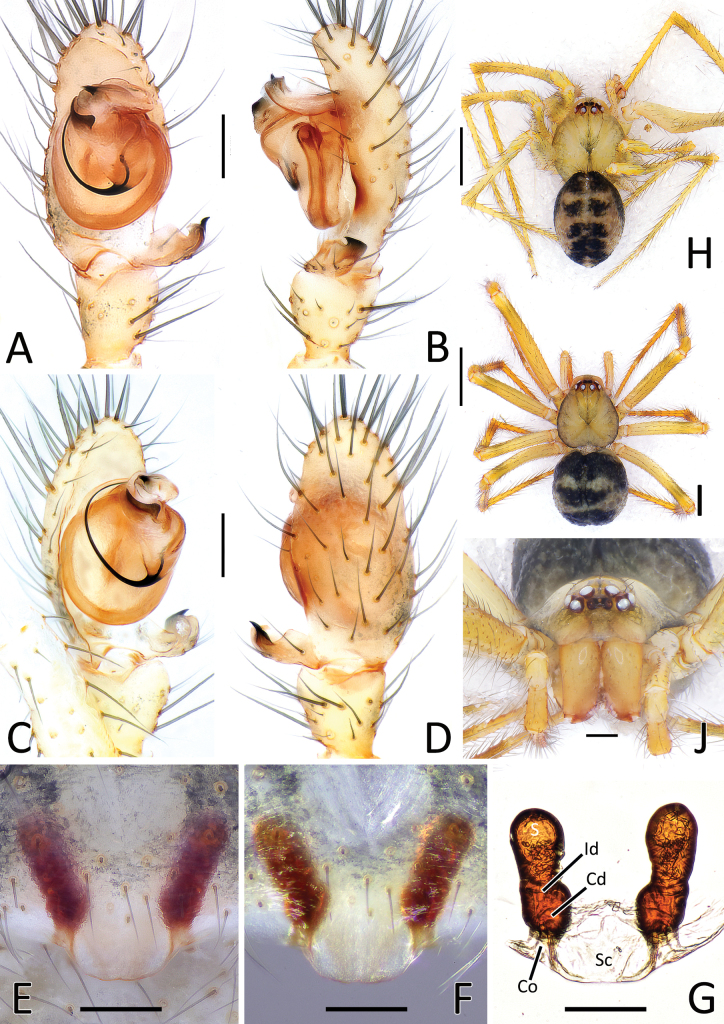
*Nesticellaterrestris***A** male palp (one of the topotypes), ventral view **B** same, retrolateral view **C** same, ventro-prolateral view **D** same, dorsal view **E** female epigyne (topotype), ventral view **F** same after dissection, shape variation **G** vulva, dorsal view **H** habitus of male (topotype) **I** habitus of female **J** cephalic area of female, frontal view. Abbreviations: Cd – copulatory duct; Co – copulatory opening; Id – insemination duct; S – spermatheca; Sc – scapus. Scale bars: 0.2 mm (**A–G, J**); 1.0 mm (**H, I**).

**Figure 7.**
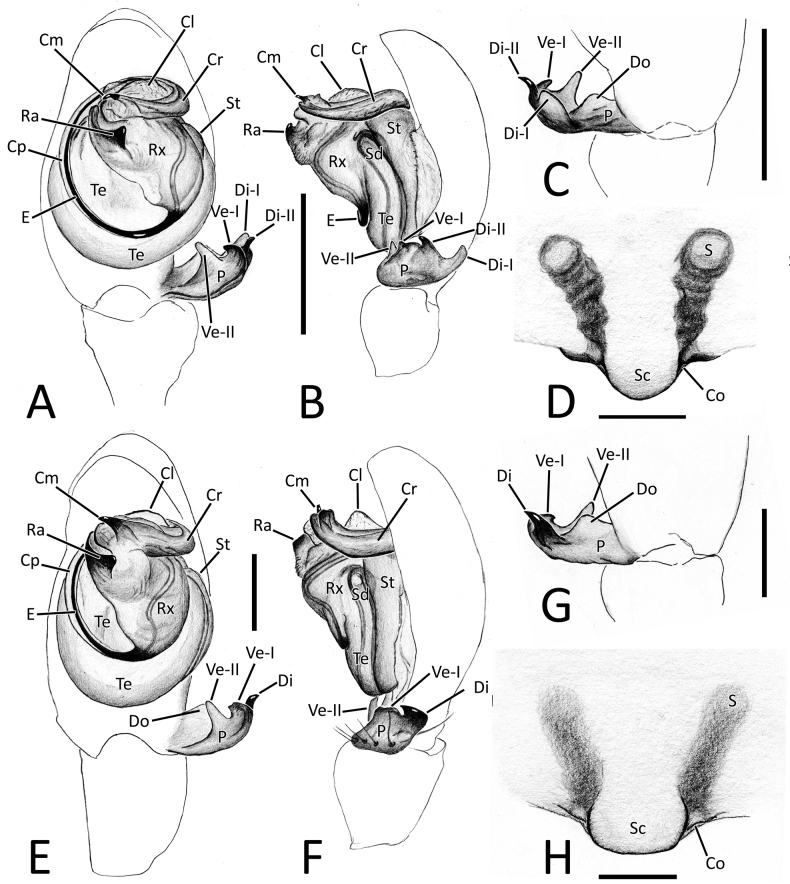
Genitalia of *Nesticellabrevipes* and *N.terrestris***A** male palp of *N.brevipes*, ventral view **B** same, retrolateral view **C** detail or paracymbium, dorsal view **E** female epigyne, ventral view **E** male palp of *N.terrestris*, ventral view **F** same, retrolateral view **C** detail or paracymbium, dorsal view **H** female epigyne, ventral view. Abbreviations: Cl – lobe of conductor; Cm – median process of conductor; Co – copulatory opening; Cp – prolateral process of conductor; Cr – retrolateral process of conductor; Di I–II – distal process(es) I and II of paracymbium; Do – dorsal process of paracymbium; E – embolus; P – paracymbium; Ra – radical apophysis; Rx – radix; S – spermatheca; Sc – scapus; Sd – sperm duct; St – subtegulum; Te – tegulum; Ve I–II – ventral processes I and II of paracymbium. Scale bars: 0.2 mm.

**Figure 8.**
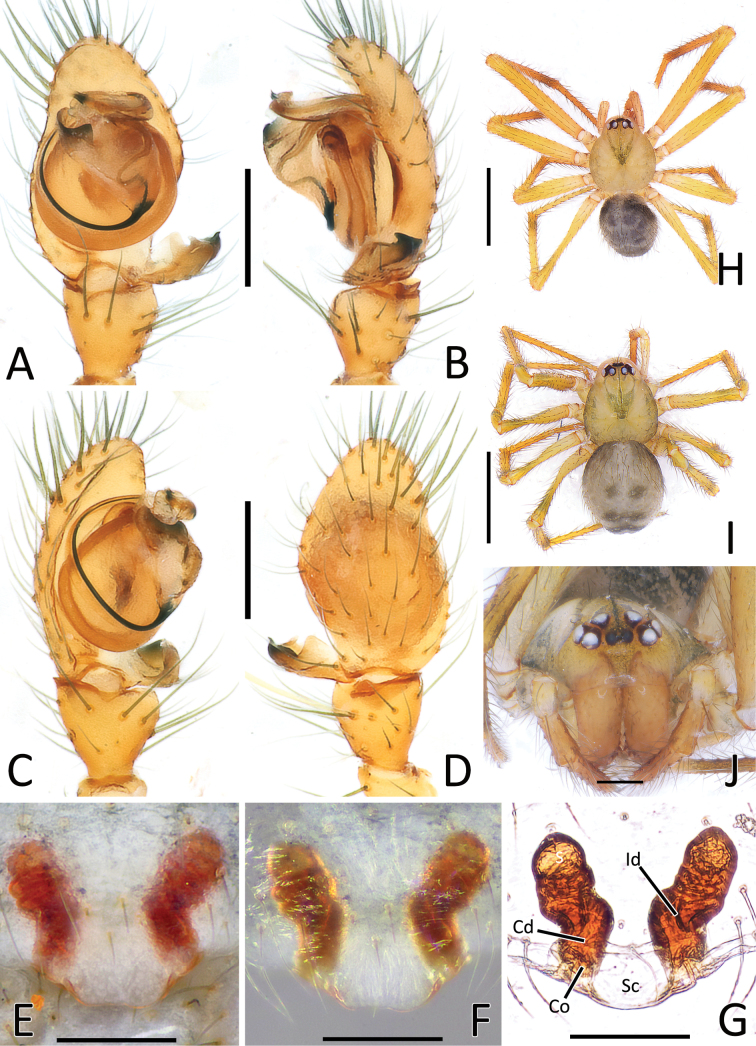
*Nesticellasilvicola* sp. nov. **A** male palp (holotype), ventral view **B** same, retrolateral view **C** same, ventro-prolateral view **D** same, dorsal view **E** female epigyne (one of the paratypes), ventral view **F** same, from shape variation **G** vulva, dorsal view **H** habitus of male **I** habitus of female **J** cephalic area of female, frontal view. Abbreviations: Cd – copulatory duct; Co – copulatory opening; Id – insemination duct; S – spermatheca; Sc – scapus. Scale bars: 0.2 mm (**A–G, J**); 1.0 mm (**H, I**).

**Figure 9.**
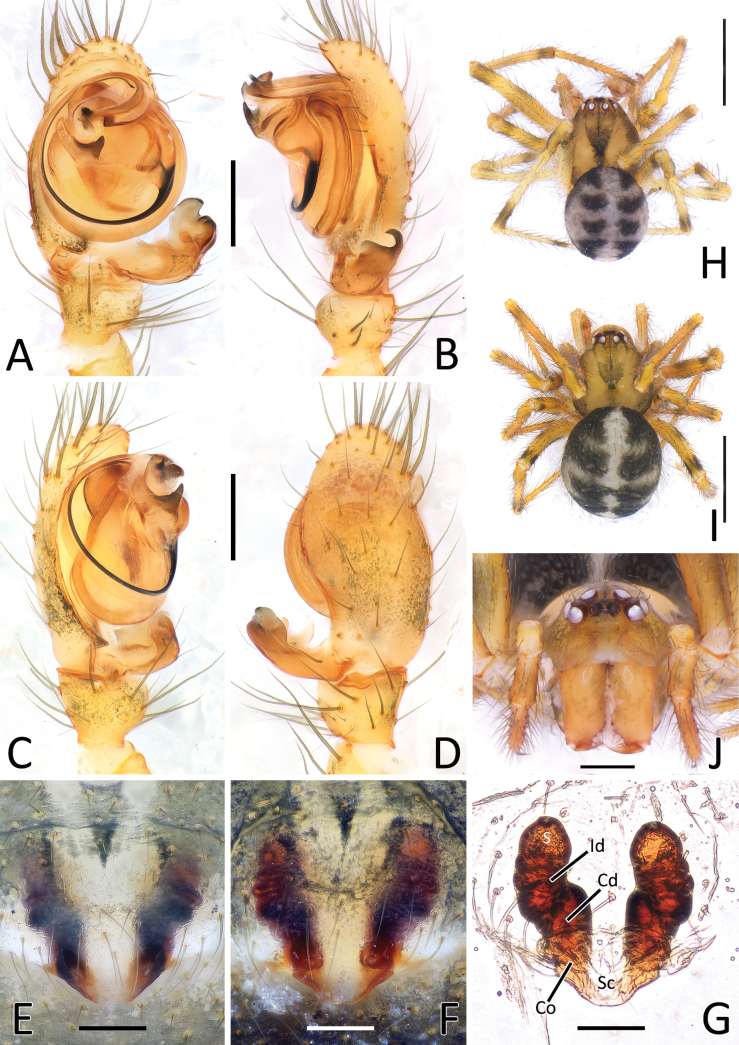
*Nesticellainsulana* sp. nov. **A** male palp (holotype), ventral view **B** same, retrolateral view **C** same, ventro-prolateral view **D** same, dorsal view **E** female epigyne (one of the paratypes), ventral view **F** same, from shape variation **G** vulva, dorsal view **H** habitus of male **I** habitus of female **J** cephalic area of female, frontal view. Abbreviations: Cd – copulatory duct; Co – copulatory opening; Id – insemination duct; S – spermatheca; Sc – scapus. Scale bars: 0.2 mm (**A–G, J**); 1.0 mm (**H, I**).

**Figure 10.**
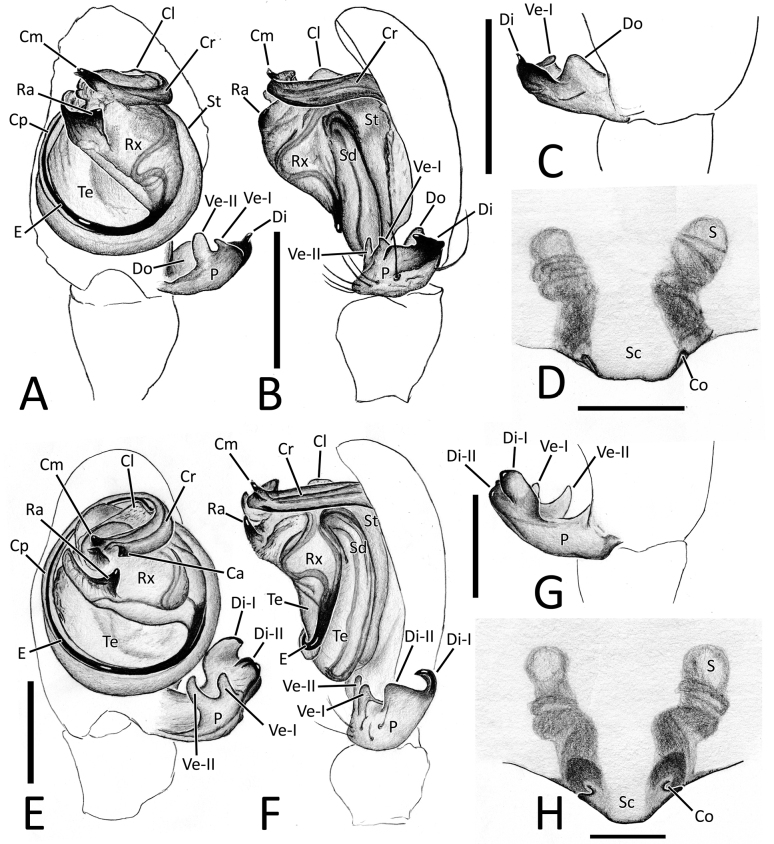
Genitalia of *Nesticellasilvicola* sp. nov. and *N.insulana* sp. nov. **A** male palp of *N.silvicola* sp. nov., ventral view **B** same, retrolateral view **C** detail or paracymbium, dorsal view **E** female epigyne, ventral view **E** male palp of *N.insulana* sp. nov., ventral view **F** same, retrolateral view **C** detail or paracymbium, dorsal view **H** female epigyne, ventral view. Abbreviations: Cl – lobe of conductor; Cm – median process of conductor; Co – copulatory opening; Cp – prolateral process of conductor; Cr – retrolateral process of conductor; Di I–II – distal process(es) I and II of paracymbium; Do – dorsal process of paracymbium; E – embolus; P – paracymbium; Ra – radical apophysis; Rx – radix; S – spermatheca; Sc – scapus; Sd – sperm duct; St – subtegulum; Te – tegulum; Ve I–II – ventral processes I and II of paracymbium. Scale bars: 0.2 mm.

**Figure 11.**
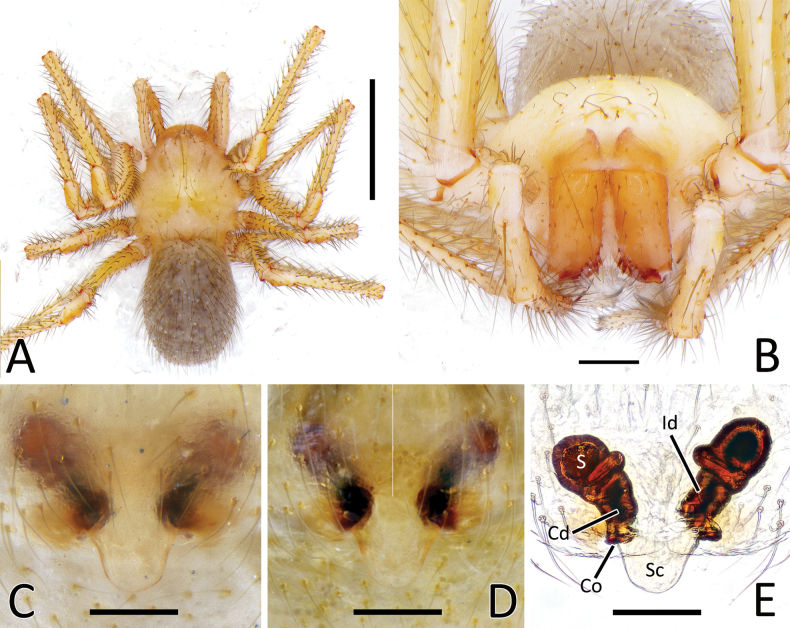
*Nesticellaocculta* sp. nov. **A** habitus of female (holotype) **B** cephalic area of female, frontal view **C** female epigyne, ventral view **D** same, shape variation **E** vulva, dorsal view. Abbreviations: Cd – copulatory duct; Co – copulatory opening; Id – insemination duct; S – spermatheca; Sc – scapus. Scale bars: 1.0 mm (**A, C**); 0.2 mm (**B, D, E**).

**Figure 12.**
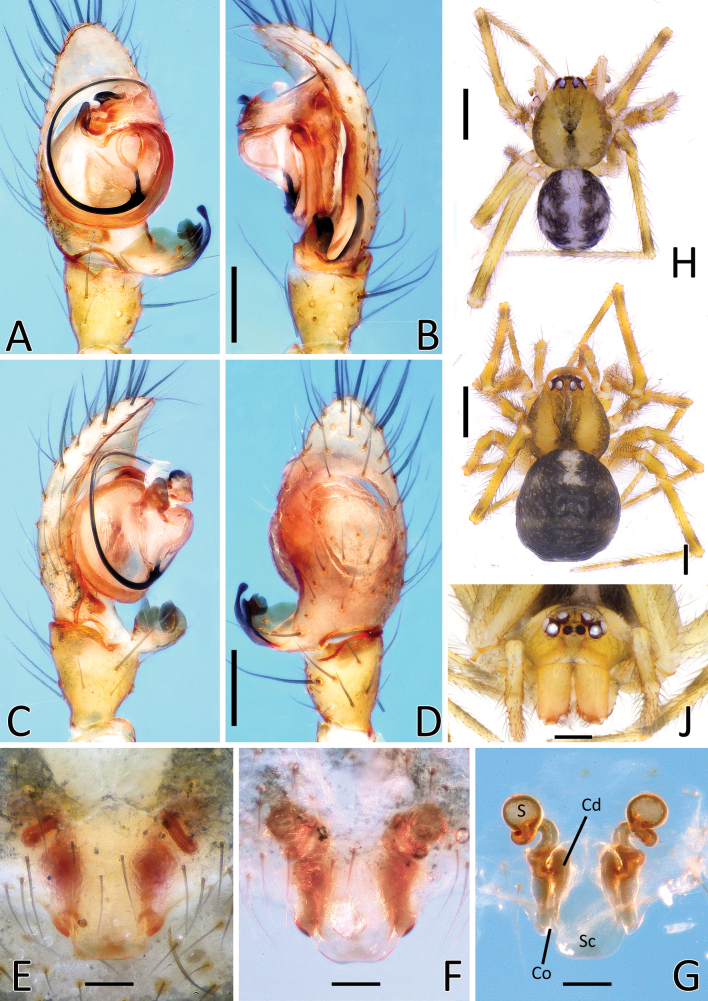
*Nesticellaokinawaensis***A** male palp, ventral view (specimen from Yambaru) **B** same, retrolateral view **C** same, ventro-prolateral view **D** same, dorsal view **E** female epigyne, ventral view (specimen from Yambaru) **F** same after dissection, shape variation (specimen from Amami-Ōshima Is.) **G** vulva, dorsal view (specimen from Yambaru) **H** habitus of male (specimen from Amami-Ōshima Is.) **I** habitus of female (specimen from Yambaru) **J** cephalic area of female, frontal view. Abbreviations: Cd – copulatory duct; Co – copulatory opening; S – spermatheca; Sc – scapus. Scale bars: 0.2 mm (**A–G, J**); 0.5 mm (**H, I**).

**Figure 13.**
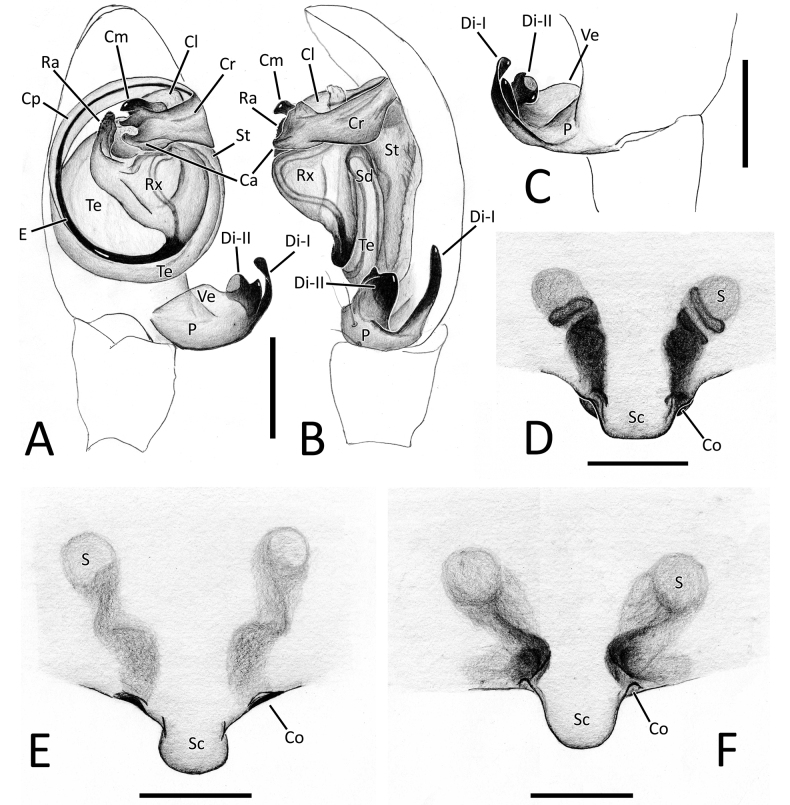
Genitalia of *Nesticellaokinawaensis*, *Howaiasubterranea* sp. nov., and *N.occulta* sp. nov. **A** male palp of *N.okinawaensis*, ventral view **B** same, retrolateral view **C** detail or paracymbium, dorsal view **D** female epigyne, ventral view; **E***H.subterranea* sp. nov., female epigyne, ventral view **F***N.occulta* sp. nov. female epigyne, ventral view. Abbreviations: Ca – apophysis of the retrolateral process of conductor; Cl – lobe of conductor; Cm – median process of conductor; Co – copulatory opening; Cp – prolateral process of conductor; Cr – retrolateral process of conductor; Di I–II – distal processes I and II of paracymbium; E – embolus; P – paracymbium; Ra – radical apophysis; Rx – radix; S – spermatheca; Sc – scapus; Sd – sperm duct; St – subtegulum; Te – tegulum; Ve – ventral process of paracymbium. Scale bars: 0.2 mm.

Based on the morphological examination of genitalia (male palp and female epigyne), we separated the studied specimens into nine morphospecies. Four are recognized as already described species (*N.brevipes*, *N.terrestris*, *N.okinawaensis*, and *H.mogera*) (Figs 1A–G, 4A–D, 5A–G, 6A–G, 7A–H, 12A–G, and 13A–D). *Nesticellaterrestris*, currently in synonymy with *N.brevipes*, shows apparent differences with the latter species in body size and shape of genitalia (Figs 5A–G, 7A–D cf. Figs 6A–G, 7E–H; see also Fig. 17A, B), suggesting its resurrection. Five additional morphospecies show unique combinations of characters that do not fit with any of the previously described taxa and are consequently described as new species (*N.insulana* sp. nov., *N.silvicola* sp. nov., *N.occulta* sp. nov., *H.alba* sp. nov., and *H.subterranea* sp. nov.) (Figs 2A–J, 3A–E, 4E–H, 8A–J, 9A–J, 10A–H, 11A–E, 13E, F).

All the species morphologically discriminated in the present study, including the undetermined morphospecies, are recovered as independent lineages with long branches. Most also have high basal node support by at least one of the two phylogenetic inferences. Such concordance provides a solid basis for considering all those morphospecies as valid in an integrative taxonomy. Among them, *N.terrestris* is also resolved as a clade clearly distinct from *N.brevipes*, thus corroborating the separation between these two species already highlighted by morphology. On the other hand, some cases require additional consideration. *Howaiamogera* is resolved as divided into two distinct, highly supported subclades (BV = 100, PP = 1), with relatively deep genetic divergence (2.5–3.7%, mean = 3.1%), in line with the results of previous phylogenies based on Chinese specimens (Zhang and Li 2013; Ballarin and Li 2018).

Similarly, species with a wide geographic distribution covering different islands show deep genetic divergences among geographically segregated populations (e.g., *N.brevipes* from Shikoku and Kyushu Is. = 7%, *N.okinawaensis* from Okinawa-honto, Kume-jima and Amami Ōshima Is. = 6–10%). Such populations appear to be separated from each other by long basal branches. Additionally, the phylogenetic relationships of some Japanese species (e.g., *N.silvicola* sp. nov., *N.brevipes*, and *N.terrestris*) remain unclear because of the low node support in the deeper parts of their branches. Nevertheless, all these three species form a highly supported clade (BV = 83, PP = 1) suggesting a common origin.

**Figure 14.**
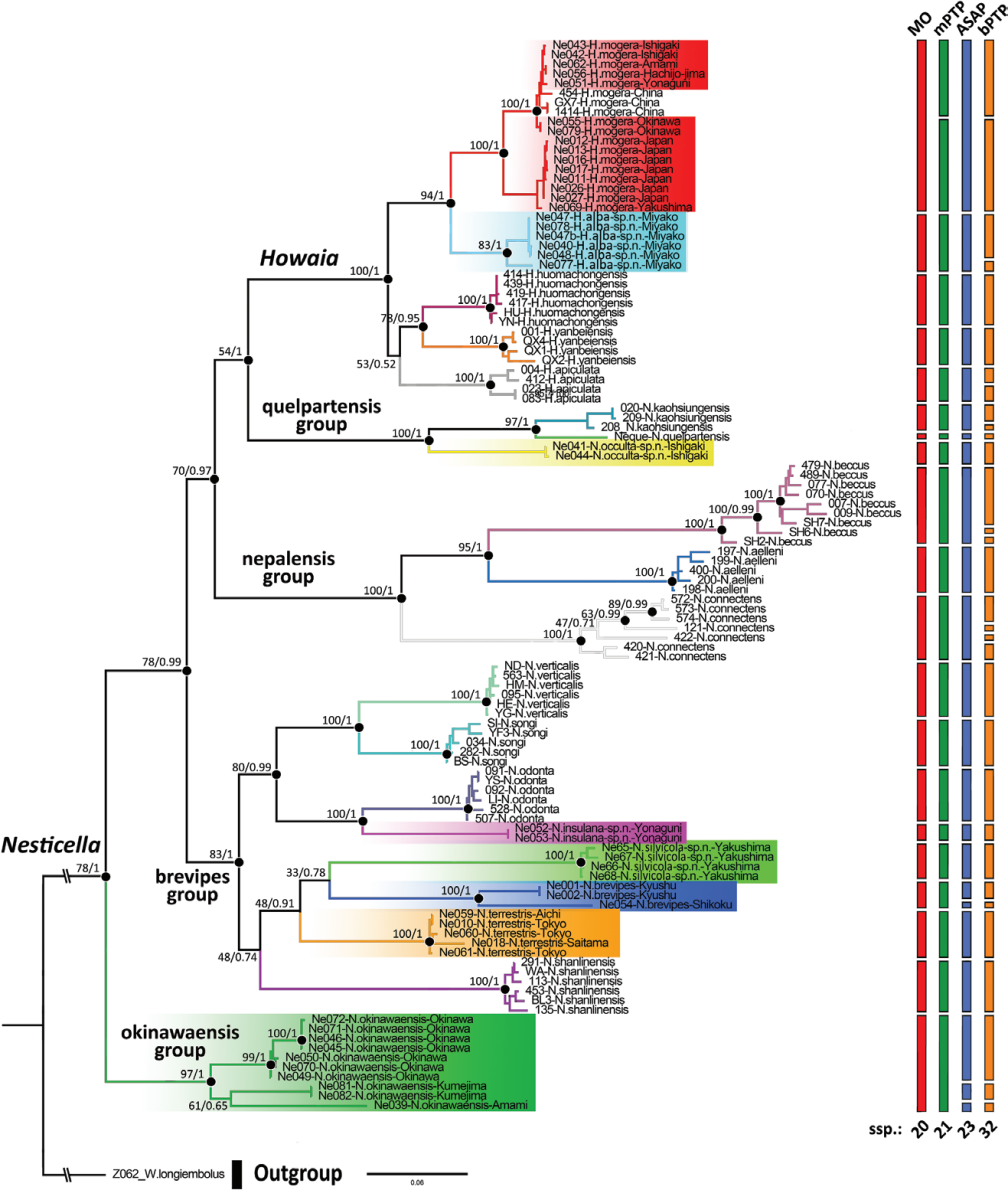
Combined phylogenetic tree based on COI gene fragment reconstructed using maximum likelihood (ML) on RAxML and Bayesian inference (BI) on MrBayes. Support at each node denotes the ML bootstrap value (BV) and BI posterior probability (PP). Nodes highly supported by at least one method (BV ≥ 70 or PP ≥ 0.95) are highlighted by a black dot. Branch lengths were scaled concerning the number of substitutions per site. Each species corresponding to monophyletic clades are represented with different branch colors, species from Japan are highlighted with a colored background: red = *H.mogera*, azure = *H.alba* sp. nov., yellow = *N.occulta* sp. nov., violet = *N.insulana* sp. nov., light green = *N.silvicola* sp. nov., blue = *N.brevipes*, orange = *N.terrestris*, dark green = *N.okinawaensis*. The tree is rooted using the species *Wraioslongiembolus*. The right lines denote the results of the species delimitation analysis based on morphological (MO) and molecular (ASAP, mPTP, and bPTP) data.

Results of the species delimitation analyses are also reported in Fig. 14 where the morphological observations are compared with the output of the ASAP, mPTP, and bPTP analyses. The mPTP analysis is highly consistent with the morphological taxonomy supporting 21 species vs 20. A slightly higher number of putative species is estimated by ASAP (23 species vs 20), while bPTP vastly exceed this number (32 vs 20) splitting into putative distinct species several morphospecies. Both *N.brevipes* and *N.okinawaensis* show a high intramorphospecific genetic divergence among geographically segregated populations, being split into more than one putative species by the ASAP and bPTP methods (but not by mPTP). In line with the morphological analysis ASAP considers the two subclades of *H.mogera* as belonging to the same species while both mPTP and bPTP split them into two putative species.

The results of the intermorphospecific pairwise-distances based on the species barcode are shown in Table 2. The interspecific genetic distance between the *Nesticella* and *Howaia* morphospecies used in this study ranges from 5.8% to 19% (mean = 14.7%), from 6.9% to 17.5% (mean = 14.7%) for the Japanese morphospecies and from 10.3% to 16.1% (mean = 14.3%) for the Japanese morphospecies excluding the three morphospecies with significant genetic structures (*H.mogera*, *N.brevipes*, and *N.okinawaensis*). Intramorphospecific diversity among the Japanese species is as follows: *N.brevipes* = 0–7.6%; *N.insulana* sp. nov. = 0%; *H.mogera* = 0–3.7%; *N.occulta* sp. nov. = 0.3%; *N.okinawaensis* = 0–7.8%; *N.silvicola* sp. nov. = 0.3–1.1%; *H.alba* sp. nov. = 0–2.5%; *N.terrestris* = 0–1.9%. We found considerable intraspecific genetic divergences in *H.mogera* (max. divergence = 3.7%), *N.brevipes* (7.6%), and *N.okinawaensis* (7.8%). This is possibly related to the strong geographic isolation of their populations inhabiting different islands. On the other hand, the genetic divergence among the allopatric populations of these species is considerably smaller than the mean of the divergence calculated among the entire species considered in this work (14.7%). It is also lesser than the minimum divergence (10.3%) among the Japanese morphospecies when these three species are excluded.

**Table 2.** Uncorrected Pairwise-distance between the Japanese and other Asian species of the genera *Nesticella* and *Howaia* based on the barcode COI partial sequence.

	*H.mogera*	H.alba sp. nov.	*N.insulana* sp. nov.	*N.brevipes*	*N.silvicola* sp. nov.	*N.terrestris*	*N.okinawaensis*	*N.occulta* sp. nov.	*N.kaohsiungensis*	*N.quelpartensis*	*H.yanbeiensis*	*H.huomachongensis*	*N.wanzaiensis*	*H.apiculata*	*N.odonta*	*N.songi*	*N.verticalis*	*N.hongheensis*	*N.shanlinensis*	*N.beccus*	*N.aelleni*	*N.connectens*	*N.yui*
***H.mogera* (Ne016)**																							
***H.alba* sp. nov. (Ne040)**	0.069																						
***N.insulana* sp. nov. (Ne052)**	0.149	0.160																					
***N.brevipes* (Ne054)**	0.157	0.155	0.141																				
***N.silvicola* sp. nov. (Ne65)**	0.153	0.149	0.136	0.129																			
***N.terrestris* (Ne061)**	0.132	0.149	0.127	0.125	0.111																		
***N.okinawaensis* (Ne046)**	0.163	0.174	0.168	0.157	0.156	0.168																	
***N.occulta* sp. nov. (Ne041)**	0.132	0.144	0.161	0.169	0.161	0.151	0.175																
***N.kaohsiungensis* (020)**	0.140	0.143	0.155	0.169	0.153	0.141	0.180	0.103															
***N.quelpartensis* (Neque)**	0.149	0.144	0.160	0.166	0.159	0.151	0.190	0.103	0.058														
***H.yanbeiensis* (QX4)**	0.101	0.108	0.146	0.160	0.141	0.144	0.166	0.149	0.160	0.157													
***H.huomachongensis* (417)**	0.089	0.097	0.147	0.158	0.144	0.144	0.171	0.138	0.151	0.147	0.065												
***N.wanzaiensis* (027)**	0.094	0.083	0.151	0.158	0.144	0.137	0.175	0.157	0.163	0.160	0.083	0.072											
***H.apiculata* (412)**	0.083	0.086	0.129	0.144	0.139	0.138	0.160	0.146	0.151	0.151	0.074	0.077	0.071										
***N.odonta* (507)**	0.141	0.151	0.094	0.144	0.132	0.108	0.160	0.149	0.155	0.151	0.146	0.147	0.141	0.140									
***N.songi* (282)**	0.134	0.152	0.108	0.131	0.127	0.106	0.149	0.149	0.147	0.158	0.141	0.151	0.138	0.137	0.103								
***N.verticalis* (563)**	0.155	0.158	0.131	0.146	0.141	0.118	0.166	0.178	0.149	0.152	0.164	0.172	0.163	0.155	0.126	0.078							
***N.hongheensis* (495)**	0.160	0.171	0.127	0.152	0.110	0.123	0.184	0.161	0.158	0.149	0.158	0.166	0.160	0.147	0.118	0.124	0.141						
***N.shanlinensis* (135)**	0.155	0.161	0.141	0.143	0.124	0.126	0.166	0.160	0.157	0.158	0.151	0.164	0.158	0.144	0.140	0.137	0.137	0.131					
***N.beccus* (SH7)**	0.154	0.155	0.158	0.178	0.169	0.147	0.166	0.146	0.140	0.155	0.169	0.158	0.154	0.160	0.157	0.161	0.158	0.172	0.158				
***N.aelleni* (400)**	0.154	0.158	0.158	0.163	0.152	0.163	0.149	0.161	0.161	0.174	0.157	0.157	0.149	0.147	0.151	0.158	0.175	0.160	0.158	0.111			
***N.connectens* (573)**	0.164	0.164	0.164	0.164	0.159	0.160	0.184	0.154	0.144	0.157	0.169	0.171	0.163	0.169	0.164	0.151	0.164	0.169	0.155	0.151	0.149		
***N.yui* (492)**	0.152	0.163	0.157	0.175	0.153	0.143	0.169	0.167	0.143	0.144	0.163	0.151	0.151	0.158	0.152	0.141	0.138	0.158	0.169	0.164	0.155	0.181	
***W.longiembolus* (062)**	0.164	0.169	0.149	0.183	0.147	0.157	0.169	0.152	0.160	0.152	0.154	0.158	0.157	0.149	0.141	0.155	0.169	0.158	0.169	0.181	0.172	0.177	0.161

As a result of the present study, we propose nine *Nesticella* and *Howaia* species for the fauna of Japan based on the combined results of morphology and molecular analyses, including five species new to science. The taxonomic revision of the Japanese species is reported in the “Taxonomic account” section. The use of a single mitochondrial gene marker for species delimitation may overestimate the number of putative cryptic species (e.g., see Hupało et al. 2022). This is probably the case with the bPTP analysis where several clades are resolved as split into putative species, often in contrast with previous taxonomic studies and with the results of the other methods used. Based on such considerations we prefer to follow a “conservative” partitioning hypothesis. Thus, in presence of inconstancy between morphological and molecular results, we used a morphology-based delimitation for the Japanese species until further studies involving multiple genetic markers and a wide number of specimens are available. However, the possible existence of further cryptic species among *H.mogera*, *N.brevipes*, and *N.okinawaensis* is not completely excluded from this conclusion. Similarly, in the case of *H.subterranea* sp. nov., we defined the species as new based on morphological comparison only due to the lack of available specimens for molecular analysis. A molecular-based confirmation of this species is thus postponed until additional fresh samples are accessible.

### ﻿Considerations on the genera *Nesticella* and *Howaia* in the Japanese and Ryukyuan archipelagos

Although Japan is considered one of the leading Asian biodiversity hotspots (Myers et al. 2000), the species richness and phylogeographic patterns of numerous epigean and subterranean arthropod taxa, including spiders (Araneae), have not yet been adequately explored. Nevertheless, new records of long-neglected taxa and the integrative use of molecular data and conventional morphology have rapidly filled this gap in recent years. The present research aims to follow this positive trend using a “modern” approach to revise in detail the state of knowledge of the genera *Nesticella* and *Howaia* in the Japanese and Ryukyuan archipelagos. Accordingly, we updated the taxonomy and distribution of the already-known species, we resurrected a previously synonymized species, and we described five additional species as new to science.

In doing so, we estimated the boundaries of the species using both morphological and molecular analyses. Our study reveals that the number of *Nesticella* and *Howaia* species in Japan is much greater than previously expected, increasing from three to nine. All the newly described species are endemic to the Ryukyus and are found on different islands. Among the new species we also report the first cases of true troglobitic *Nesticella* and *Howaia* species for the fauna of Japan. Such results further emphasize the high level of endemism of the spider fauna in the Ryukyus and are in line with other studies on Ryukyuan spiders (e.g., Xu et al. 2019). Our study also confirms that Japanese and Ryukyuan archipelagos are biodiversity hotspots for Nesticidae and subterranean spiders. Similar patterns can likely be found in other genera or families of troglophilic spiders as suggested by similar studies (e.g., Ballarin and Eguchi 2022; Suzuki et al. 2022). Special attention and collecting efforts should thus be focused on this archipelago to better explore its diversity. In this regard, the real magnitude of species diversity of the short-legged nesticid spiders in Japan is likely still not yet completely unveiled. For example, the relatively large genetic distance among conspecific individuals of *H.mogera*, *N.brevipes*, and *N.okinawaensis* suggests the possibility of genetically distinct local populations or cryptic species whose boundaries should be adequately tested in the future. Additionally, undescribed species of *Nesticella* and/or *Howaia* may still be hidden in caves or in the forest litter of poorly surveyed islands among the numerous islands forming the Japanese and Ryukyuan Archipelagos.

## ﻿Conclusions

With this research we aimed to further study the family Nesticidae in Asia toward the final goal of its comprehensive revision. We also believe that our outcomes open the door to future additional studies on Japanese and Asian nesticids concerning their diversity, historical biogeography, phylogeny, ecology, and the time and mode of island colonization and cave-adaptation. In Asia, nesticids are often endemic, locally hyperdiverse, and relatively easy to collect, and an increasing amount of data on their distribution, morphology and genetic diversity is becoming available. Thus, these spiders can be considered as a suitable model organism for revealing biogeographical and evolutionary patterns of troglophilic terrestrial arthropods (e.g., Zhang and Li 2013; Ballarin and Li 2018) and adequately detecting local hotspots of genetic diversity, a concept of increasing importance in conservation biology. In the future, additional sampling and the use of integrative studies applied to other poorly-studied nesticid genera and other “so-far-neglected” troglophilic spider taxa may help to further reveal new and interesting information on these arthropods.

## ﻿Taxonomic account

**Class: Arachnida Cuvier, 1812**

**Order: Araneae Clerck, 1757**

**Family: Nesticidae Simon, 1894**

**Tribe: Nesticellini Lehtinen & Saaristo, 1980**

### ﻿Gen. Howaia

Taxon classificationAnimaliaAraneaeNesticidae

Lehtinen & Saaristo, 1980

3832476E-5A0F-523C-A9FD-EFBF5381A5D8

= N.mogera group sensu Lin et al. 2016.

#### Type species.

*Nesticusmogera* Yaginuma, 1972 from Japan.

### ﻿ Howaia mogera

Taxon classificationAnimaliaAraneaeNesticidae

(Yaginuma, 1972)

EC1BA6FC-51FB-53DD-91D1-9C79A5126CD8

**Figure 15.**
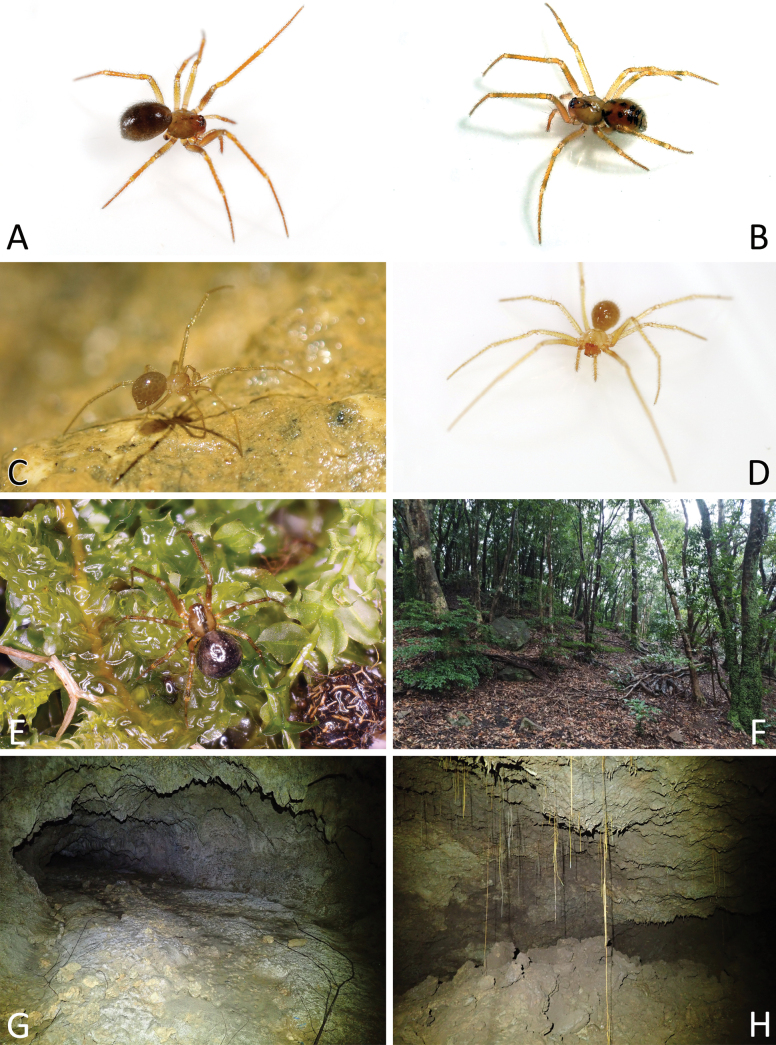
*Nesticella* and *Howaia* species in life and their natural habitats **A** female of *H.mogera***B** female of *N.terrestris***C** female of *H.alba* sp. nov. **D** female of *N.occulta* sp. nov. **E** female of *N.okinawaensis***F** example of the epigean habitat of *Nesticella* species in the Ryukyus **G** habitat of *H.alba* sp nov. (dark zone of Tsuzupisuki-abu cave) **H** habitat of *N.occulta* sp. nov. (dark zone of Kabirano-ana cave) (all photos by F. Ballarin).

**Figure 16.**
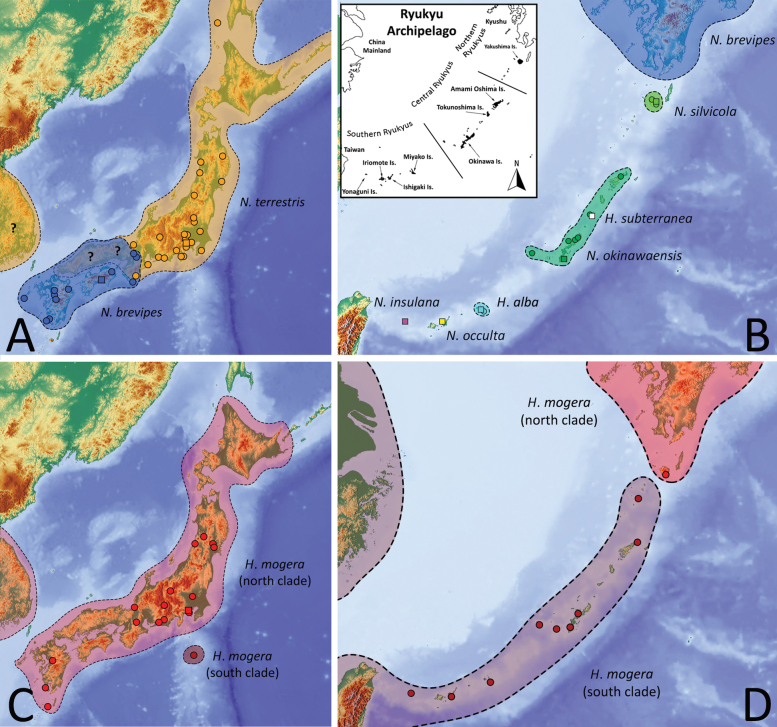
Distribution of *Nesticella* and *Howaia* species in mainland Japan and Ryukyu Archipelago **A** distribution of *N.brevipes* and *N.terrestris* in mainland Japan and neighboring countries **B** distribution of *Nesticella* and *Howaia* species endemic to the Ryukyu Archipelago **C** distribution of *H.mogera* in mainland Japan and neighboring countries **D** distribution of *H.mogera* in the Ryukyu Archipelago and neighboring countries. Colored squares refer to the type localities of the species, colored dots indicate the locations of specimens whose morphology has been checked during this study, dashed areas refer to the generally known distribution of the species, question marks refer to unclear distributions (see “remarks on misidentifications” of *N.brevipes* and *N.terrestris*).

Figs 1A–J , 4A–D , 15A , 16C, D (Japanese name: chibi-horahimegumoチビホラヒメグモ)

Nesticus terrestris Yaginuma 1970: 390, fig. 7 (♂, misidentification).
N. mogera Yaginuma 1972: 621, fig. 1 (♂♀); Yaginuma 1979: 275, pl. 6, figs 11, 12 (♂♀).
Howaia mogera Lehtinen and Saaristo 1980: 53, figs 7–9, 22–23, 29b (♂♀ transferred from Nesticus).
Nesticus brevipes Paik 1996: 72, figs 1–10 (♀, misidentification).^1^

#### Type locality.

Japan, Tokyo Pref., Tamagawa.

#### Material examined.

**Japan: Honshu Is.: Miyagi Pref.**: 1♀, Tome-gun, Lake Izunuma, 20.July.1986, A. Tanikawa leg. (MNHAH); 6♂, 4♀, Tamatukuri-gun, Naruko-cho, Myousada, 7–10.May.1996, K. Kumada leg. (NMST-Ar.3587); **Akita Pref.**: 1♀, Akita-shi, Shimokitateyanagitate, Kagawa, 21.Nov.2005, A. Fukushima leg. (NSMT-Ar.20405, identified as *N.brevipes*); **Yamagata Pref.**: 1♀, Tsuruoka-shi, Oyama, 38.75628°N, 139.76331°E, 26.Aug.2019, Y. Suzuki leg. (YSPC); **Tochigi Pref.**: 1♂ Tochigi-shi, Fujoka-machi, Uchino, Watarase-Yusuichi, 36.2353°N, 139.6574°E, Alt.: 20 m, 2.Apr.2022, N. Kikuchi leg. (FBPC); **Tokyo Pref.**: 1♂ (holotype), Tamagawa, 3.Feb.1969, H. Kobayashi leg. (NMST-Ar.73); 1♂, 2♀, Hachioji-shi, Minami-Osawa, Tokyo Metropolitan University campus, litter under bushes, 35.6245°N, 139.3863°E, 22.Sep.2020, F. Ballarin leg. (FBPC); **Hachijo-jima Is.**: 3♂, 3♀, Okago, Hachijo Botanical Park, forest litter, 33.11044°N, 139.78432°E, 03.May.2021, F. Ballarin leg. (FBPC); **Kanagawa Pref.**: 1♂, 2♀, Kawasaki-shi, Nakahara-ku, Kasugi-cho, Dec.1984 (exact date unknown), H. Ono leg. (NMST-Ar.893); 2♀, Yokohama-shi, Maioka park, 6.July.1986, A. Tanikawa leg. (MNHAH); **Nagano Pref.**: 2♀, Ueda-shi, 8.Sep.1998, Y. Fujisawa leg. (NMST-Ar.6965); 3♀, Kamiina-gun, Ijima-cho, 35.67049°N, 137.91159°E, 09.Sep.2019, Y. Suzuki leg. (YSPC); **Shizuoka Pref.**: 1♀ (Paratype), Tenryu-shi, Mar.1970 (exact date unknown), H. Kobayashi leg. (NMST-Ar.74); 1♀, Hamamatsu-shi, Kita Ward, Inasacho Okuyama, 34.85102°N, 137.62569°E, under grass tuffs in a paddy field, 30.Sep.2019, F. Ballarin leg. (FBPC); **Fukui Pref**: 1♀, Tsuruga-shi, Nagatani, under grass tuffs in a paddy field, 35.60836°N, 136.03872°E, 3.Oct.2019, F. Ballarin leg. (FBPC); **Mie Pref**: 1♀, Iga-shi, Otani, under grass tuffs in a paddy field, 34.79919°N, 136.13501°E, 7.Oct.2019, F. Ballarin leg. (FBPC); **Kumamoto Pref.**: 2♂, 1♀, Tamana-gun, Gyokutou-machi, Harakura, Yamakitasho-no-ana cave (山北小の穴), 20.May.1984, T. Irie leg. (NMST-Ar.16065); **Kagoshima Pref.**: 1♀, Minami Kyushu-shi, Kawabe-cho, 26.Feb.2007, K. Iohii(?) leg. (NMST-Ar.14585); **Yakushima Is.**: 1♂, Anbo, 207 m, broadleaf forest litter on a gentle slope 30.28458°N, 130.61799°E, 24.Sep.2021, F. Ballarin leg. (FBPC); **Suwanosejima Is.**: 1♂, 2♀, Otohime-no-dokutsu cave, 8.Jul.2022, Y. Suzuki leg. (YSPC); **Amami-Ōshima Is.**: 1♂, Amami-shi, Kasarichō Ōaza Kise, 2 m, at the base of tufts of grass on a sandy seashore, 28.46221°N, 129.65013°E, 13.Jul.2021, F. Ballarin leg. (FBPC); **Okinawa Pref.: Okinawa-Honto Is.**: 1♀, Naha-shi, Sueyoshi park, 70 m, humid broadleaves litter under trees, 26.22840°N, 127.71508°E, 21.Nov.2020, F. Ballarin leg. (FBPC); 1♂, 4♀, Nakagami-gun, Yomitan-son, Namihira, Shimuku Gama cave (シムクガマ), Alt.:72 m, large and long cave with a creek, 26.40242°N, 127.73125°E, 15.May.2022, F. Ballarin leg. (FBPC); **Aka-jima Is.**: 3♀, 17.Mar.2022, Y. Suzuki leg. (YSPC); **Kume-jima Is.**: 1♂, Shimajiri-gun, Gima, Nameless Beach, under vegetation on a sandy seashore, 26.32681°N, 126.77002°E, Alt.: 3 m, 17.May.2022, F. Ballarin leg. (FBPC); **Miyako-jima Is.**: 1♀, Nobaru Ueno, Pinza-Abu cave (ピンザアブ洞穴), 57 m, long and muddy cave, dark zone, 24.74853°N, 125.33443°E, 13.Nov.2020, F. Ballarin leg. (FBPC); **Ishigaki-jima Is.**: 4♀, Tonoshiro, Fukubukuīzā Daiichi-do cave (フクブクイーザー第1洞), 66 m, long and humid cave with a small creek, 24.36533°N, 124.17721°E, 9.Nov.2020, F. Ballarin leg. (FBPC); 4♀, same locality, 11.Nov.2020, F. Ballarin leg. (FBPC) ; **Yonaguni-jima Is.**: 1♀, Irizaki, under stones in a meadow near the seashore, 24.44499°N, 122.9411°E, 30 m a.s.l., 4.Mar.2021, F. Ballarin leg. (FBPC).

#### Diagnosis.

This species is closely related to *H.alba* sp. nov. and *H.subterranea* sp. nov. from which it can be easily distinguished by the presence of pigmentation and well-developed eyes (vs pigmentation and eyes lacking in both the other species) (Fig. 1H–J cf. Figs 2H–J, 3A–C). Males of *H.mogera* can also be separated from males of *H.alba* sp. nov. by the thinner paracymbium (P) and the more squared and stockier distal process of paracymbium (Di) (vs wider P and longer and slightly sharper Di in *H.alba* sp. nov.) (Figs 1A, B, D, 4A–C cf. Figs 2A, B, D, 4E–G). Females of *H.mogera* are distinguished from females of *H.alba* sp. nov. and *H.subterranea* sp. nov. by the shape of scapus (Sc), rectangular and with a flat posterior margin (vs stockier and larger Sc in *H.alba* sp. nov. or longer and distally dilatated Sc in *H.subterranea* sp. nov., both having a rounded posterior margin) (Figs 1E–G, 4D cf. Figs 2E–G, 3D, E, 4H, 13E). See also Lin et al. (2016) for the diagnosis of *H.mogera* with other congeners of the same species group.

#### Redescription of male

**(holotype).** (redescription of habitus based on freshly collected specimen from Tokyo area due to the discoloration of the holotype). Habitus as in Fig. 1H. Total length. 1.94, Prosoma 1.10 long, 0.89 wide. Carapace rounded, yellowish with slightly darker striae. Cervical groove and fovea distinct. Eyes well developed. Eyes measurements: AME = 0.06, ALE = 0.09, PME = 0.09, PLE = 0.09, AME–ALE = 0.03, ALE–PLE = 0.00. Chelicerae, labium, maxillae, and sternum of same color as carapace. Legs uniformly yellowish. Legs measurements: I 5.07 (1.41, 0.43, 1.30, 1.24, 0.69), II 3.85 (1.10, 0.38, 0.90, 0.89, 0.58), III 3.26 (0.97, 0.33, 0.73, 0.73, 0.50), IV 4.44 (1.29, 0.40, 1.11, 1.06, 0.58). Opisthosoma uniformly dark grey with slightly lighter mark on antero-dorsal side.

Male palp as in Figs 1A–D, 4A–C. Cymbium relatively elongated, 3–4 robust spines on distal-prolateral margin (Fig. 1D). Paracymbium with 1 distal (Di) and 1 ventral (Ve) processes. Distal process short, squared, and stocky, distinctly sclerotized. Ventral process sharp, spine-like, headed internally (Figs 1A–D, 4A–D). Embolus (E) long and filiform, origin of embolus positioned at ~ 6:00 o’clock on radix (Rx). Radical apophysis (Ra) broad and dorsally flat with a granulate surface. Conductor with 3 distinct processes (Cp, Cr, Cm) and a half-transparent distal lobe (Cl). Prolateral process (Cp) flat and long, ribbon-like, headed counterclockwise, wrapped around the embolus. Retrolateral process of conductor (Cr) wide and thick, curved internally, with a broadened, flat central part. Median process of conductor (Cm) strongly sclerotized, short and stout, horn-like, ending with a blunt tip and having a smaller, stout ventral process (Figs 1A–C, 4A, B).

#### Redescription of female

**(based on specimen from Tokyo).** Habitus as in Figs 1I, 15A. Total length: 2.44, Prosoma 1.19 long, 0.98 width. Cephalic area as in Fig. 1J. Carapace piriform. Eyes measurements: AME = 0.05, ALE = 0.08, PME = 0.08, PLE = 0.08, AME–ALE = 0.04, ALE–PLE = 0.01. Legs measurements: I 4.93 (1.42, 0.44, 1.28, 1.15, 0.64), II 3.96 (1.08, 0.39, 0.85, 0.78, 0.54), III 2.88 (0.86, 0.33, 0.63, 0.62, 0.44), IV 4.22 (1.30, 0.41, 1.10, 0.89, 0.52). Coloration and other details as in male.

Epigyne and vulva as in Figs 1E–G, 4D. Scapus (Sc) elongated antero-posteriorly, rectangular, slightly longer than wide, ending with a flat posterior margin (Figs 1E–G, 4D). Copulatory opening (Co) at the inner-lateral sides of scapus. Internal ducts slightly visible through the transparent tegument, V-shaped. Copulatory ducts (Cd) straight, short, and thick, gradually diverging from each other, slightly coiled in the first half of the trait before reaching the spermathecae. Insemination ducts (Id) thin, coiled around the copulatory ducts. Spermathecae (S) small and rounded, separated from each other by ~ 1.5 their diameter (Fig. 1G).

#### Size variation.

Male (based on 5 specimens): total length: 1.89–2.02, prosoma length: 0.84–1.07, prosoma width: 0.76–0.85. Female (based on 10 specimens): total length: 2.08–2.88, prosoma length: 0.96–1.15; prosoma width: female: 0.87–0.96.

#### Distribution.

East Asia (from South China to Korea and Japan). Introduced to Europe, Azerbaijan, and several oceanic islands (Hawaii, Fiji, Reunion, St. Helena, etc.). Although it is likely that *H.mogera* is naturally distributed in Asia, its precise center of origin, as well as the time and mode of its dispersion outside the Asian continent, are still unstudied. In Japan the species is widespread along the whole country in both mainland Japan and the Ryukyus (Fig. 16C, D).

#### Habitat and ecology.

This species has a broad environmental tolerance. In Japan *H.mogera* can be found in a wide range of habitats, both natural and artificial, including caves, mines, artificial tunnels, small animal burrows, forest leaf litter, humid meadows, paddy fields, marshes, urban parks, greenhouses, vegetated seashores, coastal environments, etc. *Howaiamogera* usually builds simple scaffold webs in external habitats, under superficial stones, in empty spaces among the leaf litter or at the base of tufts of grass. Apparently, the populations living in mainland Japan are found less commonly in caves or cave-like habitats. In contrast, in addition to epigean environments, the populations living in the Ryukyu islands can be found more frequently in natural subterranean habitats, dwelling in both the twilight and dark areas of caves and tunnels.

#### Remarks on intraspecific variation.

Coloration and pattern of the opisthosoma can be rather variable, depending on the population or individual. Usually, populations living in mainland Japan show a darker habitus with an opisthosoma uniformly black or dark grey, sometimes having one or few small lighter marks on the dorsal side (Figs 1H, I, 15A). Instead, individuals living in the Ryukyus seems to show a lighter pattern bearing several larger greyish dorsal marks, often merged together forming a continuous median stripe. Such pattern is shared by the southern Chinese populations (see Liu and Li 2013: fig. 18B). Legs are usually uniformly yellowish; however, some individuals show a faint darker annulation on the distal part of femur and tibia. The shape of scapus can also be slightly variable among individuals or populations, ranging from strongly rectangular to slightly inverted-trapezoidal or shorter and more squared, more rarely with strongly rounded distal margin (e.g., see Fig. 1E, F and Liu and Li 2013: fig. 18C, D). Yaginuma (1972: 620) illustrated a scapus with a strongly triangular shape for the paratype of *H.mogera*. Such extreme shape is abnormal and never observed by us in any of the examined specimens. In addition, the drawing by Yaginuma does not perfectly match with the shape of the scapus of the original sample (paratype NMST-Ar.74 from Tenryu-shi) which shows a normal rectangularly-shaped scapus.

#### Remarks on misidentifications.

*Howaiamogera* was initially misidentified by Yaginuma (1970) and described as the male of *Nesticus* (= *Nesticella*) *terrestris* based on a specimen from Tamagawa, Tokyo. Soon after Yaginuma recognized the mistake and described the species as new based on the same male together with a female specimen from Shizuoka (Yaginuma 1972). Until now *H.mogera* has been recorded in several different countries and redescribed and illustrated numerous times by different authors (World Spider Catalog 2023). However, these descriptions were all based on specimens collected far from the type locality, in different Asian countries (e.g., Korea: Kim et al. 1999, Kim and Lee 2018; China: Gong and Zhu 1982, Liu and Li 2013) or based on introduced populations (e.g., Fiji Is.: Lehtinen and Saaristo 1980; Hawaii Is.: Gertsch 1984; Azeirbaijan: Marusik and Guiseinov 2003; Poland: Bielak-Bielecki and Rozwalka 2011; Italy: Pantini et al. 2020). Herein, for the first time after the original description, we illustrate and redescribe the holotype and additional specimens from the type locality area.

In the past, the lack of information about the taxonomy of *H.mogera*, and in general on *Nesticella* species, has been the cause of misidentifications by senior arachnologists, sometimes confusing this species with other similar congeners. For example, the male of the blind *H.mogera* specimens from Miyako-jima Is. illustrated by Shimojana (1977) refers to the newly described *H.alba* sp. nov. Paik (1996) recorded *Nesticus* (= *Nesticella*) *brevipes* from South Korea based on female specimens. The illustrated samples do not match the morphology of this species (cf. Figs 5I, E–G, 7D and Paik 1996: figs 1–10) and probably they refer to more than one species of *Nesticella* or *Howaia*. Among them, the shape of epigyne and internal ducts of the specimens of the so-called groups A or B fits well with those of *H.mogera* (Paik 1996: figs 6, 9, 10). Illustration of the female of *H.mogera* by Zhu and Zhang (2011: fig. 34A–C), clearly do not refer to this species, the illustrated epigyne and vulva being morphologically different from those found in the genus *Howaia*.

#### Remarks on phylogeny and biogeography.

Previous molecular analyses suggest that populations of *H.mogera* in Eastern Asia group into two well-distinct subclades with non-overlapping distribution, distributed respectively in North-Eastern Asia (= north clade) and South China (= south clade) (Zhang and Li 2013; Ballarin and Li 2018; this work). In Japan both these two clades are apparently present. The north clade is distributed in mainland Japan covering the islands of Hokkaido, Honshu, Shikoku, and Kyushu. Its southernmost boundary seems to correspond to the island of Yakushima (Fig. 16C). The south clade shares the same genetic pattern of the southern China populations (Fig. 14) and it is widespread along the whole Ryukyus and in the island of Hachijo-jima Is., south of Tokyo (Fig. 16C, D). The presence of the south clade in Hachijo-jima Is., far away from the other known records, suggests a possible artificial introduction to this island. Some degrees of genetic difference (2.5–3.7%) and slight variations in the body pattern and habitat preference can be observed in populations belonging to the two clades (see remarks on habitat and variation discussed above). Nevertheless, no clear distinct morphological differences are observed in their genitalia. The result of our species delimitation analysis only partially supports them as two distinct species (Fig. 14). It is possible that they represent two cryptic species or, more likely, an early stage of species differentiation which is still in progress nowadays.

### ﻿ Howaia alba sp. nov.

Taxon classificationAnimaliaAraneaeNesticidae

1F03361C-B84A-5821-AA11-3CFAA248A60E

https://zoobank.org/140385CD-7B9B-421C-9752-7182907C5369

Figs 2A–J , 4E–H , 15C , 16B (Japanese name: tsuzupisuki-horahimegumo ツヅピスキホラヒメグモ)

Nesticella mogera Shimojana 1977: 353, fig. 6 (♂, misidentification).

#### Type material.

♂ ***Holotype*** (NMST-Ar. 25251): **Japan: Okinawa Pref.: Miyako-jima Is.**: Shimozato Hirara, Oharaminami Park, Tsuzupisuki-abu cave (ツヅピスキアブ), 32 m, long and humid cave, in the dark zone of the cave, 24.79468°N, 125.28192°E, 12.Nov.2020, F. Ballarin leg.

***Paratypes*: Japan: Miyako-jima Is.**: 3♀, same data as the holotype (NSMT-Ar 25252); 1♀, same locality 14.Nov.2020, F. Ballarin leg. (RMUF); 5♀, same locality, 16.Sep.2022, F. Ballarin leg. (2♀ MNHAH, 3♀ FBPC); 4♀, Nobaru Ueno, Pinza-abu cave (ピンザアブ洞穴), 57 m, long and muddy cave, in the dark zone of the cave, 24.74853°N, 125.33443°E, 13.Nov.2020, F. Ballarin leg. (RMUF); 3♀, same locality, 17.Sep.2022, F. Ballarin leg. (FBPC).

#### Other material examined.

**Japan: Miyako-jima Is.**: 1 juv., Nakabari, Nakabari Limestone Cave (仲原鍾乳洞), 24.73384°N, 125.37610°E, 29.Dec.2021, R. Miyata leg. (FBPC).

#### Etymology.

The specific name is derived from the Latin word for the color white (*albus*, adjective) referring to the whitish coloration of the species.

#### Diagnosis.

The new species is similar to *H.mogera* and to the troglobitic species *H.rongtangensis* (Lin, Ballarin & Li, 2016) from Hainan Island, *H.subterranea* sp. nov., and *N.occulta* sp. nov. Male of *H.alba* sp. nov. can be distinguished from male of *H.mogera* and *H.rongtangensis* by the different shape of the larger paracymbium (P) bearing a longer, slimmer, and sharper distal process (Di) (vs slimmer P with a shorter, larger, and blunter Di in *H.mogera* and *H.rongtangensis*) (Figs 2A–D, 4E–G cf. Figs 1A–D, 4A–C and Lin et al. 2016: fig. 44A, B, D). Female of the new species are distinguished from female of *H.mogera*, *H.subterranea* sp. nov., and *N.occulta* sp. nov. by the larger and stockier scapus (Sc) with a slightly rounded posterior margin (vs slimmer Sc with a flat posterior margin in *H.mogera*, a longer Sc with a wider lobated tip in *H.subterranea* sp. nov., and a slimmer, tongue-like Sc ending with a strongly concave tip in *N.occulta* sp. nov.) (Figs 2E–G, 4H cf. Figs 1E–G, 3D, E, 4D, 11C, D, 13E, F).

#### Description of male

**(holotype).** Habitus as in Fig. 2H. Total length 1.88. Prosoma 0.94 long, 0.83 wide. Carapace uniformly pale yellowish. Eyes completely degenerated and reduced to white maculae. Cervical groove and fovea indistinct. Chelicerae brownish. Labium, maxillae, and sternum of the same pale color as carapace. Legs uniformly pale yellowish. Leg formula: I, IV, II, III. Legs measurements as follows: I 6.17 (1.61, 0.47, 1.60, 1.72, 0.77), II 4.71 (1.32, 0.39, 1.20, 1.18, 0.62), III 3.89 (1.11, 0.31, 0.95, 1.02, 0.50), IV 5.17 (1.50, 0.37, 1.34, 1.31, 0.65). Opisthosoma uniformly greyish-yellow, covered with long, sparse hairs.

Male palp as in Figs 2A–D, 4E–G. Cymbium relatively elongated, covered with thin sparse setae, bearing some thicker setae on the distal-prolateral margin (Fig. 2D). Paracymbium with a single distinctly sclerotized, stocky distal process (Di), slightly elongated near the tip and a single sharp, spine-like ventral process (Ve) (Figs 2A–D, 4E–G). Embolus (E) long and filiform, origin of embolus positioned at ~ 6:00 o’clock on radix (Rx). Radical apophysis (Ra) broad, with a granulate surface. Conductor with 3 distinct processes (Cp, Cr, Cm) and a half-transparent distal lobe (Cl). Prolateral process of the conductor (Cp) flat, ribbon-like and headed counterclockwise, wrapped around embolus. Retrolateral process of conductor (Cr) wide and thick, curved internally, with a broadened, flat central part. Median process of conductor (Cm) stout, horn-like, strongly sclerotized bearing a smaller, stout ventral process. (Figs 2A–C, 4E, F).

#### Description of female

**(one of the paratypes).** Habitus as in Figs 2I, 15C. Total length 2.3. Prosoma 1.09 long, 0.92 wide. Cephalic area as in Fig. 2J. Coloration and other details as in male. Legs measurements as follows: I 6.56 (1.83, 0.50, 1.74, 1.67, 0.82), II 5.09 (1.46, 0.42, 1.27, 1.22, 0.72), III 4.09 (1.31, 0.36, 0.90, 0.92, 0.60), IV 5.46 (1.65, 0.45, 1.42, 1.27, 0.67).

Epigyne and vulva as in Figs 2E–G, 4H. Scapus (Sc) short and stumpy, approximately as long as wide, ending with a slightly rounded posterior margin (Figs 2E, F, 4H). Copulatory opening (Co) at the inner-lateral sides of scapus. Internal ducts slightly visible through the transparent tegument, shaped as a narrow V. Copulatory ducts (Cd) short and thick, slightly divergent to each other, slightly twisted in the inner trait with 1 coil, curving outward and then inward before reaching the spermathecae. Insemination ducts (Id) thin, coiled around the Cd). Spermathecae (S) small and rounded, separated from each other by ~ 2× their diameter (Fig. 2G).

#### Size variation.

Female (based on 5 specimens): total length: 2.00–2.67, prosoma length: 1.05–1.12, prosoma width: 0.92–0.97.

#### Distribution.

Endemic to Miyako-jima Is., Ryukyus, Japan (Fig. 16B).

#### Habitat and ecology.

*Howaiaalba* sp. nov. is found in the natural caves in Miyako-jima Is. This species builds simple scaffold webs between rocks and in crevices at the base of the walls or on the floor of the caves. It dwells exclusively in the dark zone of the caves, in areas characterized by relatively high and uniform temperature and humidity (e.g., Tsuzupisuki-abi cave: temp: 25.2 °C, hum: 94.1%; Pinza-Abu cave: temp: 25.1 °C, hum: 92.6%) (Fig. 15G). Adults of *Howaiaalba* sp. nov. were observed preying on Schizomida (*Bamazomussiamensis* (Hansen, 1905) which roam the floor of the caves in Miyako-jima Is. Females carrying the eggs cocoon attached to their spinnerets were also observed (but not collected) sitting on webs or wandering under rocks. Despite extensive surveying, no specimens were found in the numerous artificial tunnels or underground water reserves dug in the limestone rocks of the island. The complete absence of eyes and pigmentation, the lack of external records and the finding of the species only in the deepest areas of the caves identify *H.alba* sp. nov. as a true troglobiont.

#### Remarks on misidentifications.

This species was recorded and illustrated for the first time by Shimojana (1977: fig. 6A–C). Due to the general similarities in the shape of genitalia, it was identified as *H.mogera* although the author highlighted the lack of eyes in these specimens (Shimojana 1977: 353). Our analysis, based on both morphology and molecular data, supports *H.alba* sp. nov. as a closely related species but clearly distinct from *H.mogera*.

### ﻿ Howaia subterranea sp. nov.

Taxon classificationAnimaliaAraneaeNesticidae

A35A96E4-C38B-5886-9857-DC213176118F

https://zoobank.org/A2EE02AE-8523-43F8-A888-AE2F3061459D

Figs 3A–E , 13E , 16B (Japanese name: kaiken-horahimegumo カイケンホラヒメグモ)

#### Material examined.

♀ ***Holotype*: Japan: Kagoshima Pref.: Okinoerabu-jima Is.**: 1♀, Kaikendo cave (海見洞), 3.May.2004 H. Tamura leg. (NSMT-Ar 25253).

#### Etymology.

The new species is named after the Latin adjective *subterraneus* (= underground, subterranean). It refers to the troglobitic lifestyle of this species.

#### Diagnosis.

This species is similar to *H.mogera* and the other troglobiont species *H.alba* sp. nov. and *N.occulta* sp. nov. *Howaiasubterranea* sp. nov. can be distinguished from these species by the different shape of the epigyne, having curved internal ducts (vs straight ducts in the other three species), and a longer scapus (Sc) with an enlarged tip (vs a shorter and more rectangular Sc with a flat distal margin in *H.mogera*, a shorter and stockier Sc in *H.alba* sp. nov., and a shorter, tongue-like Sc in *N.occulta* sp. nov., all of them lacking a clearly enlarged tip) (Figs 3D, E, 13E cf. Figs 1E–G, 2E–G, 4D, H). In addition, the new species can be easily distinguished from *H.mogera* and the other Japanese species by the lack of eyes and pigmentation (vs present in the other troglophilic congeners).

#### Description.

**Female (holotype).** Habitus as in Fig. 3A, C. Total length 2.65. Prosoma 1.20 long, 0.93 wide. Carapace piriform, uniformly pale yellowish. Eyes strongly degenerated, reduced to white maculae (Fig. 3B). Cervical groove and fovea indistinct. Chelicerae uniformly brownish. Labium, maxillae, and sternum pale yellowish as carapace. Legs uniformly pale yellowish. Legs measurements (leg III missing): I 6.73 (1.95, 0.46, 1.82, 1.65, 0.85), II 5.24 (1.56, 0.45, 1.29, 1.22, 0.72), III (-), IV 5.75 (1.76, 0.46, 1.54, 1.32, 0.67). Opisthosoma uniformly greyish, covered by long, sparse hairs.

Epigyne and vulva as in Figs 3D, E, 13E. Scapus (Sc) elongated antero-posteriorly, ~ 2× longer than wide, ending with an enlarged, lobated tip (Figs 3D, 13E). Copulatory opening (Co) at the inner-lateral sides of scapus. Internal ducts slightly visible through the transparent tegument, shaped as a narrow curly bracket. Copulatory ducts (Cd) bent in middle trait, first trait slightly curved outward then curving anteriorly before reaching spermathecae (Figs 3D, E, 13E). Insemination ducts (Id) thin, coiled around the copulatory ducts. Spermathecae (S) small and rounded, separated from each other by ~ 2.5× their diameter (Fig. 3D, E).

**Male.** Unknown.

#### Distribution.

Endemic to Okinoerabu-jima Island. Known only from the type locality (Fig. 16B).

#### Habitat and ecology.

*Howaiasubterranea* sp. nov. has probably been collected in the dark zone of the type locality cave. The lack of pigmentation and the strongly reduced eyes further suggest this species as a true troglobiont. Nevertheless, the lack of specimens and additional information do not allow us to define in detail the ecology and micro-habitat preference of this species as well as its precise phylogenetic position. Nevertheless, the morphology of epigyne clearly identify *H.subterranea* sp. nov. as belonging to the genus *Howaia*.

### ﻿Gen. Nesticella

Taxon classificationAnimaliaAraneaeNesticidae

Lehtinen & Saaristo, 1980

AA5E0B12-E443-5B2D-9301-B12AADAD8FF2

#### Type species.

*Nesticusnepalensis* Hubert, 1973 from Nepal.

##### ﻿*Nesticellabrevipes* group

### ﻿ Nesticella brevipes

Taxon classificationAnimaliaAraneaeNesticidae

(Yaginuma, 1970)

0131E743-1547-5FFF-9147-8ADB3F1A7D01

Figs 5A–J , 7A–D , 16A (Japanese name: ko-horahimegumo コホラヒメグモ)

Theridion pilula Komatsu 1940: 194, fig. 5a–d (♀, misidentification).
Nesticus brevipes Yaginuma 1970: 386, figs 1, 2 (♂); Yamaguchi and Yaginuma 1971: 172, figs 1, 2 (♀); Yaginuma 1972: 619, fig. 2 (♂♀); Irie 1981: 31, figs 1–3 (♂♀); Chikuni 1989: 45, fig. 3 (♂♀); Kamura and Irie 2009: 353, fig. 106 (♀).

#### Type locality.

Japan Shikoku Is., Kochi Pref., Tosa-Yamada-cho, Sakagawa, Ryuga-dō cave (龍河洞).

#### Material examined.

**Japan: Honshu Is.: Wakayama Pref.**: 1♀, Higashimuro-gun, Kushimoto, 23.Aug.1993, A. Tanikawa leg. (FBPC); **Shiga Pref.**: 1♀, Koga, Shigaraki-cho, Miyajiri, 28.Jun.2022, M. Yoshida leg. (FBPC); 1♀, Otsu, Sakamoto, 23.Jul.2022, M. Yoshida leg. (FBPC); **Shikoku Is.: Tokushima Pref**: 2♀, Tokushima, Nyūtachō, Konji, Konjiji temple (建治寺), narrow and dry tunnel in the cliff near the temple, 34.02769°N, 134.42923°E, 13.May.2019, F. Ballarin leg. (FBPC); **Kochi Pref.**: 1♀ (holotype), Kami, Tosayamadacho Sakakawa, Ryuga-dō cave (龍河洞), 11.Apr.1970, S. Ueno leg. (NMST-Ar.75); 1♂ (paratype), same data and locality (NMST-Ar.76); **Kyushu Is.: Saga Pref.**: 1♀, Fujicho, 31.Jul.2005, A. Tanikawa leg. (MNHAH); **Kumamoto Pref.**: 1♂, 3♀, Aso-gun, Aso-machi, Kikuchi Keikoku gorge, 8.Aug.2003, T. Irie leg. (NSMT-Ar.5689); same locality, 11.Jul.2004, T. Irie leg. (NSMT-Ar.5713); 1♂, 2♀, Kami-mashiki-gun, Tonochi-machi, Kashiwagawa, 23.May.2004, T. Irie leg. (NMST-Ar.5728, identified as *H.mogera*); 1♀, Kuma-gun, Itsuki-mura, Otaki, 13.May.2004, T. Irie leg. (NMST-Ar.5721); **Ōita Pref.**: 2♀, Ōita-shi, Ochi Shimohetsugi, Ōzuru-doukustu-iseki cave (尾津留洞窟遺跡), 38 m large and rather dry cave, 33.16747°N, 131.67679°E, 17.Mar.2019, F. Ballarin leg. (FBPC); **Kagoshima Pref.**: 2♀, Minami Kyushu-shi, Kawabe-cho, 26.Feb.2007, K. Ishii leg. (NMST-Ar.14585, identified as *H.mogera*); 1♀, Minamisatsuma-shi, Kasasa-cho; 9.Dec.2007, K. Ishii leg. (NSMT-Ar.14513).

#### Diagnosis.

This species is closely related to *N.terrestris* and *N.silvicola*. Male of *N.brevipes* can be distinguished from male of the latter two species by the presence of two distal processes of paracymbium (Di-I–II), a sharper radical apophysis (Ra), and a thinner median process of conductor (Cm) (vs a single, sharper Di, a stockier Ra, and a thicker Cm in *N.terrestris* and *N.silvicola*). (Figs 5A–D, 7A–C cf. Figs 6A–D, 7E–G, 8A–D, 10A–C). In addition, the origin of the embolus (E) from the radix is located in a different position than in *N.terrestris* (4:30 o’clock in *N.brevipes* vs 6:00 o’clock in *N.terrestris*) (Figs 5A, 7A cf. Figs 6A, 7E).

**Figure 17.**
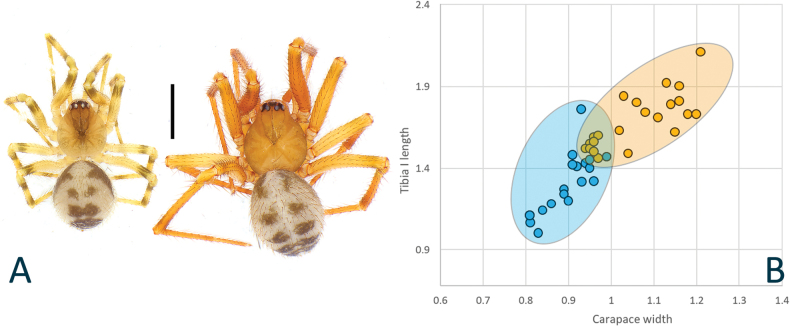
Size comparison between *Nesticellabrevipes* and *N.terrestris***A** visual comparison of adult females of *Nesticellabrevipes* (left) and *N.terrestris* (right) **B** Scatterplot of the Tibia I / carapace lengths ratio in females of *N.brevipes* (blue circle) and *N.terrestris* (orange circles). Measurements are reported in millimeters. Scale bar: 1 mm (**A**).

Female of *N.brevipes* can be separated from female of *N.terrestris* and *N.silvicola* by the slimmer scapus (Sc), approximately as long as wide, usually with a more rounded posterior margin (vs a larger Sc, ~ 1.5–2.0× wider than long, having a flatter posterior margin in *N.terrestris* and *N.silvicola*) (Figs 5E, F, 7D cf. Figs 6E, F, 7H, 8E, F, 10D). In addition, *N.brevipes* shows wider spermathecae (S) than the diameter of the copulatory ducts (Cd) (vs same diameter of Cd in *N.terrestris* and *N.silvicola*) and internal ducts with a more convoluted and irregular trend (vs more straight ducts in *N.terrestris* or strongly bent in the middle in *N.silvicola* both with less clear coils). (Figs 5E–G cf. Figs 6E–G, 8E–G). In addition, *N.brevipes* is generally smaller is size than *N.terrestris* (females 1.76–2.50 vs 2.31–3.30, see also Fig. 17A, B).

#### Redescription of male

**(paratype).** Habitus as in Fig. 5H. Total length 1.94. Prosoma 1.02 long, 0.87 wide. Carapace rounded, uniformly brown-yellowish with borders and central area slightly darker. Cervical groove and fovea distinct. Chelicerae, labium, maxillae, and sternum of the same uniform color as carapace. Eyes well developed. Eyes measurements: AME = 0.03, ALE = 0.06, PME = 0.07, PLE = 0.07, AME–ALE = 0.04, ALE–PLE = 0.00. Legs uniformly pale yellowish. Legs measurements: I 7.28 (2.04, 0.47, 1.92, 2.04, 0.81), II 5.70 (1.70, 0.43, 1.44, 1.45, 0.68), III 4.23 (1.30, 0.38, 0.96, 1.00, 0.59), IV 5.56 (1.86, 0.43, 1.51, 1.18, 0.58). Opisthosoma greyish with large black marks on dorsal and frontal sides.

Male palp as in Figs 5A–D, 7A–C. Cymbium relatively short, covered with sparse setae, several thicker setae on distal-prolateral margin (Fig. 5D). Paracymbium with 2 hook-like distal processes (Di-I–II), 2 ventral processes (Ve-I–II), and a single dorsal apophysis (Do). Distal process I (Di-I) thick, headed antero-retrolaterally; distal process II (Di-II) slimmer and sharper, headed retrolaterally; ventral process I (Ve-I) short and stocky, headed internally; ventral process II (Ve-II) long and lobated, headed internally; dorsal apophysis (Do) lobated, wide and flat (Figs 5A–D, 7A–C). Embolus (E) long and filiform, origin of embolus positioned at ~ 4:30 o’clock on radix (Rx). Radical apophysis (Ra) strongly sclerotized, triangular with a rather sharp tip. Conductor with 3 distinct processes (Cp, Cr, Cm) and a half-transparent distal lobe (Cl). Prolateral process of the conductor (Cp) flat, ribbon-like, headed counterclockwise and wrapped around embolus. Retrolateral process of conductor (Cr) wide and thick, curved inside. Median process of conductor (Cm) tiny and slim, strongly sclerotized with a ribbon-like lobe wrapped around its prolateral side (Figs 5A–C, 7A, B).

#### Redescription of female

**(holotype).** Habitus (fresh specimen from Shikoku) as in Fig. 5I. Total length 2.24. Prosoma 1.02 long, 0.92 wide. Cephalic area as in Fig. 5J. Carapace piriform. Eyes measurements: AME = 0.02, ALE = 0.06, PME = 0.05, PLE = 0.06, AME–ALE = 0.05, ALE–PLE = 0.00. Coloration and other details as in male. Legs yellowish with darker annulation on femur and tibia. Legs measurements as follows: I 6.55 (1.88, 0.46, 1.74, 1.66, 0.81), II 5.07 (1.52, 0.40, 1.24, 1.21, 0.70), III 3.85 (1.20, 0.30, 0.85, 0.91, 0.59), IV 5.23 (1.67, 0.43, 1.31, 1.17, 0.65).

Epigyne and vulva as in Figs 5E–G, 7D. Scapus (Sc) short and stumpy, approximately as long as wide, ending with a rounded, convex posterior margin (Figs 5E, F, 7D). Copulatory opening (Co) at the inner-lateral sides of scapus. Internal ducts slightly visible through the transparent tegument, shaped as a narrow V. Copulatory ducts (Cd) short, straight, and thick, slightly divergent from each other. Insemination ducts thin, coiled around the copulatory ducts. Spermathecae (S) rounded, slightly wider than copulatory ducts, separated from each other by ~ 2× their diameter (Fig. 5G).

#### Size variation.

Male (based on 3 specimens): total length: 1.94–2.31, prosoma length: 1.02–1.17, prosoma width: 0.87–0.99. Female (based on 10 specimens): total length: 1.76–2.50, prosoma length: 0.90–1.15, prosoma width: 0.81–0.99.

#### Distribution.

Western Japan (Shikoku, Kyushu, western Honshu?), China? (Fig. 16A). The easternmost limit of this species in Japan seems to be located in the Kansai area where it apparently overlaps with the distribution of *N.terrestris* (Fig. 16A). The precise boundaries between these two species remain unclear. All samples and published drawings of *N.brevipes* from China, Korea, and Russian Far East checked by us refer to different species of *Nesticella*. In lack of clear records, the presence of this species outside Japan remains unconfirmed although it seems unlikely. See also “remarks on misidentifications” for additional information.

#### Habitat and ecology.

*Nesticellabrevipes* can be found in humid and shadowed environments such as undergrowth of deciduous and evergreen forests growing in narrow valleys, vegetated cliffs, screes, caves, and artificial tunnels. This species builds simple scaffold webs in empty spaces among the leaf litter, between rocks or in the crevices in the walls and on the floor of caves. In subterranean environments we collected this species in both the twilight and the dark zones.

#### Remarks on intraspecific variation.

Like many other nesticid species *N.brevipes* shows a certain degree of intraspecific variation in the shape of genitalia and in particular in the shape of the scapus of the female epigyne. Some individuals from Shikoku Is. (e.g., from Kikuchi Keikoku Gorge), and especially populations from the Kansai area, show a more squared scapus with the posterior margin more flattened than normal. A high degree of intraspecific genetic divergence (7.6%) is also observed between the population living in Shikoku Is. and Kyushu Is.

#### Remarks on misidentifications.

*Nesticellabrevipes* was first described and illustrated by Komatsu (1940) based on specimens from Ryuga-do cave in the island of Shikoku but wrongly identified as *Theridionpilula* (= *Phoroncidiapilula* (Karsch, 1879), Fam. Theridiidae Sundevall, 1833). Yaginuma (1970: p. 386–388, figs 1, 2) recognized the species as new to science and described it based on specimens from the same cave. Nevertheless, he misidentified the male of the closely related *N.terrestris* thus reporting under *N.brevipes* a mix of specimens from the two species (Yaginuma 1970: p. 388, 390). Two years later Yaginuma (1972: p. 619–621), in his revision of short-legged nesticids from Japan, synonymized *N.terrestris* with *N.brevipes* although it recognized them as belonging to different morpho-groups based on the morphology of the male palp and female epigyne. The outcome of our study, based on both morphological and molecular evidence, clearly supports the original separation of *N.brevipes* and *N.terrestris* as two distinct species.

Since the time of its description *Nesticellabrevipes* “sensu lato” has been frequently recorded by Japanese or foreign authors, in Japan and in other surrounding countries (World Spider Catalog 2023, see Shinkai et al. 2022 for the Japanese records). Nevertheless, due to the previous synonymization of *N.terrestris* with *N.brevipes*, it is difficult to understand to which species these records refer without directly checking the genitalia of the specimens. Thus, in this work we considered only the records of *N.brevipes* which samples have been directly examined by us or of which clear published drawings of genitalia were available. Based on the examined material we confirm the presence of *N.brevipes* in the island of Kyushu and Shikoku and in the Kansai area. We could not check any specimen from the Chugoku area thus the presence of this species in the western part of the Honshu Island, although possible, still needs to be properly confirmed. All records from central-eastern Honshu examined by us refer instead to the similar *N.terrestris* (see Fig. 16A). Yaginuma (1970, 1972) cited the presence of an unusual population of *N.brevipes* inhabiting some caves in Mie Prefecture (e.g., Fubonji-do cave). These specimens show partially reduced eyes (Yaginuma 1970: fig. 10) and, apparently, morphological characters of palp and epigyne mixed between those of *N.brevipes* and *N.terrestris* (Yaginuma 1972: p. 620, fig. 3). We did not have the opportunity to examine these specific specimens; however, other samples collected in both epigean and hypogean environments in Mie Pref. and checked by us refer to the similar *N.terrestris*. It is possible that the *Nesticella* from Fubonji-do cave represent a different and still undescribed troglobitic species. Irie (1981), in his work on cave spiders from Kyushu, illustrate a population of *N.brevipes* from the Kikuchi Keikoku Gorge, northern Kumamoto Pref., which female show a wide and squared scapus (Irie 1981, figs 2, 3). These specimens were examined by us and, although some individuals have the scapus more squared and sometimes wider than usual, both males and female show the diagnostic characters of *N.brevipes*.

Concerning the records outside Japan, specimens of *N.brevipes* from Kuril Is. (Marusik and Crawford 2006) were inspected by us and they refer to *N.terrestris*. We did not have the possibility to check samples from Korea, nevertheless none of the published drawings of Korean specimens show the diagnostic characters of *N.brevipes*. Based on the shape of the epigyne they clearly refer to *H.mogera* (cf. Figs 1A–G, 4A–D vs Paik 1996: figs 6, 9, 10) or to other species of *Nesticella* of the *N.brevipes* group, including possibly *N.terrestris* (cf. Figs 5A–G, 7A–D vs Figs 6A–G, 7E–H vs Paik 1996: figs 7, 8 vs Namkung 2002: fig. 80a, b vs Kim and Lee 2018: fig. 27b, c). On the basis of these observations, we consider the presence of *N.brevipes* in Korea unlikely.

During his previous studies, the first author had the occasion to examine several *Nesticella* specimens from China identified as *N.brevipes* and preserved in the collections of the Chinese Academy of Sciences, Beijing. All these specimens were revealed to be misidentifications of other endemic *Nesticella* or *Howaia* species. For example, the specimens determined as *N.brevipes* by Zhang and Li (2013) were recognized by the first author as *H.huomachongensis* (Lin, Ballarin & Li, 2016). The drawing of the male palp of *N.brevipes* from Zhejiang, China, published in Song et al. (1999) do not allow a clear identification of the species, it may refer to *N.brevipes* as well as another similar Chinese species of the *N.brevipes* group which was not yet described at the time of the publication of the book. Considering such circumstances, the presence of *N.brevipes* in China is unlikely but needs to be confirmed.

### ﻿ Nesticella terrestris

Taxon classificationAnimaliaAraneaeNesticidae

(Yaginuma, 1970)

4248F014-D99B-5403-9CBB-DE7554492492

Figs 6A–J , 7E–H , 15B , 16A (Japanese name: azuma-ko-horahimegumo アズマコホラヒメグモ)

Nesticus terrestris Yaginuma 1970: 390, figs 3–6, 8 (♀) (described ♂ refers to H.mogera).
N. brevipes Yaginuma 1970: fig. 9 (♂, misidentification); Yamaguchi and Yaginuma 1971: 172, figs 1, 2 (♀, misidentification); Yaginuma 1972: 619, figs 3(?), 4 (♂♀, synonymized); Yaginuma 1977: 315, pl. 2, fig. 18 (♂); Yaginuma 1979: 275, pl. 6, figs 9, 10 (♂♀); Yaginuma 1986: 55, fig. 29.7 (♂♀); Namkung 2002: 80, fig. 12.4a, b (♂♀) (?); Kamura and Irie 2009: 353, figs 103–105, 107 (♂♀); Marusik and Kovblyuk 2011: 199, fig. 25.1, 2, 4, 5 (♂♀); Kim and Lee 2018: 26, fig. 11A–C (♂♀) (?).
Howaia brevipes Marusik and Crawford 2006: 187, figs 20, 32–33 (♂♀).

#### Type locality.

Japan, Tokyo Pref., Mt. Kagenobuyama.

#### Material examined.

**Japan: Honshu Is.: Iwate Pref.**: 1♀, Iwate-gun, Kuzumaki-machi, 4.Aug.1995, H. Okawa leg. (NSMT-Ar.11299, identified as *N.brevipes*); **Miyagi Pref.**: 1♂, 1♀, Minamisanriku-cho, Mt. Tatsugane, 21.July.2013, A. Tanikawa leg. (MNHAH); **Akita Pref.**: 1♀, Akita-shi, Shimokitateyanagitate, Akahira, 18.Jul.2005, A. Fukushima leg. (NSMT-Ar.17417, identified as *N.brevipes*); **Yamagata Pref.**: 3♀, Oguni-machi, Tamagawanakazato, 18.July.2010, A. Tanikawa leg. (MNHAH); 2♀, Nukumidaira, 18.July.2010, A. Tanikawa leg.; 1♀, Kotamagawa, 19.July.2010, A. Tanikawa leg. (MNHAH); 1♀, Sakata-shi, Tamasudarenotaki Waterfall, 38.99706°N, 140.05332°E, 27.Aug.2019, Y. Suzuki leg. (YSPC); **Ibaraki Pref.**: 1♀, Tsukuba-shi, Oda, Mt. Hokyo-san, 36.15260°N, 140.11853°E, 22.Aug.2017, Y. Suzuki leg. (YSPC); 1♂, 1♀, Tsukuba-shi, Mt. Tsukuba, 36.22662°N, 140.09885°E, 29.Jun.2019, Y. Suzuki leg. (YSPC); **Tochigi Pref.**: 1♀, Imaichi-shi, Iwasaki, 12.Aug.1990, A. Tanikawa leg. (MNHAH); 1♀, Nikko-shi, 7.Jul.1996, Y. Suganami leg. (NSMT-Ar.17205, identified as *N.brevipes*); **Saitama Pref.**: 1♂, 2♀; Iruma-gun, Motoyama, near Kamakita cave, Aza-ana cave, 11.Aug.1974, leg. Unknown (FBPC); 3♂, Hiki-gun, Ogawa-machi, Kami-furutera, Furutera-do cave, 6.Jul.2019, T. Hiramatsu leg. (FBPC); 1♂, 6♀, Chichibu-shi, Kuroya, Iwane-do cave, 36.055145°N, 139.114867°E, 06.Nov.2022, T. Nagai leg. (TNPC); **Chiba Pref**: 1♂, Kamogawa-shi, Kiyosumi, 35.13982°N, 140.17725°E, 20.Feb.2020, Y. Suzuki leg. (YSPC); **Tokyo Pref.**: 1♀ (holotype), Mt. Kagenobuyama, 20.Dec.1968, E. Shinkai leg. (epigyne dissected and not present in the vial) (NMST-Ar.72); 1♂, 4♀ (topotypes, ~ 3 km Est of the type locality area of the species), Hachioji-shi, Hachioji, trail near the Hachioji castle ruins, 339 m, scree in a narrow valley near a temporary creek, 35.64654°N, 139.25175°E, 31.May.2021, F. Ballarin leg. (FBPC); 1♂, 1♀, Hachioji-shi, Mt. Jinba, 27.May.1984, H. Ono leg. (NSMT-Ar.13401, identified as *N.brevipes*); 1♀, Okutama-machi, Kaniwasawa, 35.83906°N, 139.07345°E, 27.Jun.2020, Y. Suzuki leg. (YSPC); 1♀, Nishitama-gun, Nippara, near Ichiishiyama Shrine, 1015 m, leaf litter in a beech forest, 35.85506°N, 139.03513°E, 5.May.2022, F. Ballarin leg. (FBPC); **Kanagawa Pref.**: 1♂, Kawasaki-shi, Tama-ku, Ikuta Ryokuchi, 29.Jun.1991, M. Ban leg. (NSMT-Ar.10062, identified as *N.brevipes*); 1♀, Atsugi-shi, Nanasawa, 17.Apr.1997, M. Ban leg. (NSMT-Ar.10814, identified as *N.brevipes*); **Fukui Pref.**: 1♀, Onyu-gun, Natashou, Nagatani, 30.Jul.2002, K. Kumada leg. (NSMT-Ar.15214, identified as *N.brevipes*); **Shizuoka Pref.**: 1♂, 8♀, Tagata-gun, Amagiyugashima-cho, Mt. Ichiyama, 14.Feb.1983, K. Kumada leg. (NSMT-Ar.17762, identified as *N.brevipes*); 2♀, Kamo-gun, Higashiizu, Mt. Amagi, 18.Oct.1986, A. Tanikawa leg. (MNHAH); 2♀, Fujinomiya-shi, Myojoyama Park, 17.Nov.2014, A. Tanikawa leg. (MNHAH); 1♀, Fujinomiya-shi, Hitoana, Shin-ana Cave (新穴), long and humid lava cave (temp: 11.7 °C, hum: 95.3%), 35.36406°N, 138.59478°E, 725 m, 3.Dec.2022, F. Ballarin leg. (FBPC); 2♀, Hamamatsu-shi, Tenryu-ku, Ryokukeidai, 34.86801°N, 137.79494°E, 2–3.Jan.2018, Y. Suzuki leg. (YSPC); 1♀, Susono-shi, Iwanami, Iwanami Fuketsu Wind Cave (岩波風穴), warm and humid lava cave (temp: 18.9 °C, hum: 99.9%), 35.21835°N, 138.92003°E, 255 m, 2.Dec.2022, F. Ballarin leg. (FBPC); **Aichi Pref.**: 1♀, Toyohashi-shi, Suse-cho, near the entrance of Susenoja-ana cave (嵩山蛇穴), 34.79678°N, 137.48575°E, litter of a mixed forest, 29.IX.2019, F. Ballarin leg. (FBPC); **Mie Pref.**: 2♀, Ise-shi, Ujitachi-cho, Naigu, Ise Jingu shrine, 8.Nov.2003, K. Kumada leg. (NSMT-Ar.14059); 1♂, 1♀, Kiya, Koumori-ana (こうもり穴), 27.Sep.–10.Oct.1980, Y. Terumi leg. (OMNH); **Russia: Sakhalin Oblast: Moneron Is.**: 1♂, 1♀, 23.Aug.2001, Y.M. Marusik leg. (YMPC, identified as *N.brevipes*).

#### Diagnosis.

This species is closely related to *N.brevipes* and *N.silvicola*. But generally larger in size than the latter two species (females 2.31–3.30 vs 1.76–2.50 in *N.brevipes* and 1.84–1.94 in *N.silvicola*, see also Fig. 17A, B). Male of *N.terrestris* can be distinguished from male of *N.brevipes* by the presence of a single, sharper distal process of paracymbium (Di), a stockier radical apophysis ending with a rounded tip (Ra), and a thicker median process of the conductor (Cm) (vs two Di, a sharper Ra ending with a pointy tip, and a thinner Cm in *N.brevipes*). (Figs 6A–D, 7E–G cf. Figs 5A–D, 7A–C). It can be separated from male of *N.silvicola* by the sharper distal process of paracymbium (Di), the stockier and rounder radical apophysis (Ra), and by the thinner ventral process II of paracymbium (Ve-II), (vs stockier Di, sharper Ra, and wider Ve-II in *N.silvicola*). (Figs 6A–D, 7E–G cf. Figs 8A–D, 10A–C). In addition, the origin of the embolus I from the radix is located in a different position than in the other two species (6:00 o’clock in *N.terrestris* vs 4:30 o’clock in *N.brevipes* and *N.silvicola*) (Figs 6A, 7E cf. Figs 5A, 7A, 8A, 10A).

Female of *N.terrestris* can be separated from female of *N.brevipes* by the thicker and more squared scapus ~ 1.5–2× longer than wide and having a flat posterior margin (vs a slimmer scapus approximately as long as wide, with a rounded posterior margin in *N.brevipes*) (Figs 6E, F, 7H cf. Figs 5E, F, 7D). In addition, *N.terrestris* shows spermathecae (S) which diameter is approximately as wide as the copulatory ducts (vs S wider than copulatory ducts in *N.brevipes*) and straight internal ducts with a regular trend and few visible coils (vs ducts with a more irregular trend and more visible coils in *N.brevipes*). (Fig. 6E–G cf. Fig. 5E–G). Female of *N.terrestris* can be separated from female of *N.silvicola* by the more lobated scapus (Sc) with rounder lateral margins and straight internal ducts (vs more squared lateral margins and strongly bent internal ducts in *N.silvicola*) (Figs 6E–G, 7H cf. Figs 8E–G, 10D).

#### Description of male

**(one of the topotypes).** Habitus as in Fig. 6H. Total length 2.54. Prosoma 1.22 long, 1.18 wide. Carapace rounded, uniformly yellowish with borders and central area slightly darker. Cervical groove and fovea distinct. Eyes well developed. Eyes measurements: AME = 0.06, ALE = 0.09, PME = 0.09, PLE = 0.09, AME–ALE = 0.06, ALE–PLE = 0.01. Chelicerae, labium, maxillae, and sternum of the same color as carapace. Legs yellowish with darker annulation on femur, patella, and tibia. Legs measurements as follows: I 7.07 (2.03, 0.47, 1.99, 1.77, 0.81), II 5.56 (1.64, 0.47, 1.41, 1.31, 0.73), III 4.55 (1.39, 0.41, 1.09, 1.07, 0.59), IV 6.12 (1.89, 0.47, 1.61, 1.48, 0.67). Opisthosoma greyish with large black marks on dorsal and lateral sides.

Male palp as in Figs 6A–D, 7E–G. Cymbium relatively short, covered with sparse setae, several thicker setae on distal-prolateral margin (Fig. 6D). Paracymbium with a single distal process (Di), two ventral processes (Ve-I–II), and a dorsal apophysis (Do): distal process (Di) thick, hook-like when observed laterally, with a sharp tip headed ventrally and retrolaterally; ventral process I (Ve-I) stocky and blunt, headed internally; ventral process II (Ve-II) long, headed internally; dorsal apophysis (Do) lobated, wide and flat (Figs 6A–D, 7E–G). Embolus (E) long and filiform, origin of embolus positioned ~ 6:00 o’clock on radix (Rx). Radical apophysis (Ra) strongly sclerotized, triangular, ending with a rounded tip. Conductor with 3 distinct processes (Cp, Cr, Cm) and a half-transparent distal lobe (Cl). Prolateral process of the conductor (Cp) flat, ribbon-like and headed counterclockwise, wrapped around embolus. Retrolateral process of conductor (Cr) wide and thick, curved inside. Median process of conductor (Cm) thick and strongly sclerotized, spine-like, ending sharp, with a ribbon-like lobe wrapped around its prolateral side. (Figs 6A–C, 7E, F).

#### Redescription of female

**(one of the topotypes).** Habitus as in Fig. 6I, 15B. Total length 2.95. Prosoma 1.55 long, 1.16 wide. Cephalic area as in Fig. 6J. Carapace piriform. Eyes measurements: AME = 0.06, ALE = 0.09, PME = 0.09, PLE = 0.09, AME–ALE = 0.07, ALE–PLE = 0.00. Coloration and other details as in male. Legs measurements as follows: I 7.85 (2.32, 0.61, 2.13, 1.91, 0.88), II 6.01 (1.79, 0.53, 1.52, 1.41, 0.76), III 4.72 (1.46, 0.44, 1.04, 1.11, 0.67), IV 6.65 (2.19, 0.53, 1.72, 1.47, 0.74). Epigyne and vulva as in Figs 6E–G, 7H. Scapus (Sc) short and stumpy, rectangular, laterally elongated, ~ 1.5–2.0× wider than long, ending with a flat or slightly curved posterior margin (Figs 6E, F, 7H). Copulatory opening (Co) at the inner-lateral sides of scapus. Internal ducts slightly visible through the transparent tegument, V shaped. Copulatory ducts (Cd) short, straight, and thick, strongly diverging from each other. Insemination ducts thin, coiled around the Cd. Spermathecae (S) small and rounded, separated from each other by ~ 2–2.5× their diameter (Fig. 6G).

#### Size variation.

Male (based on 5 specimens): total length: 2.01–2.54, prosoma length: 1.05–1.25, prosoma width: 0.95–1.18. Female (based on 15 specimens): total length: 2.31–3.30, prosoma length: 1.03–1.45, prosoma width: 0.94–1.21.

#### Distribution.

Japan (Hokkaido, central-eastern Honshu, western Honshu?), Russian Far East (Sakhalin and Kuril Is.), Korea? (Fig. 16A). The presence of this species in western Honshu and Korea needs confirmation. See also “remarks on misidentification” for additional notes on the distribution of *N.terrestris*.

#### Habitat and ecology.

*Nesticellaterrestris* is found in humid and shadowed habitats, including forest leaf litter, under rotten logs and stones, vegetated cliffs, screes, and caves, both limestone and lava caves. This species builds simple scaffold webs in empty spaces among leaf litter and in rock recesses.

#### Remarks on intraspecific variation.

This species shows some degree of variation in size and in the shape of the epigyne, different individuals having a slightly wider or slightly narrower scapus. The posterior margin is usually straight but it might be also slightly concave or slightly convex depending by the individuals. Specimens from the Kansai area seem to have a general smaller size and a scapus proportionally narrower than those of other populations.

#### Remarks on misidentifications.

*Nesticellaterrestris* was originally described by Yaginuma (1970: p. 387, 390, 391, figs 3–5) on the basis of a female collected from the Tokyo area. The author misidentified the male describing a male of *H.mogera* as a paratype of *N.terrestris* (Yaginuma 1970: p. 387, 391, fig. 8). An additional male from Gifu was also wrongly identified as *N.brevipes* and illustrated but not described (Yaginuma 1970: p. 387, fig. 9). The other records reported in the same work are probably a mix of the three species (Yaginuma 1970: p. 392). *Nesticellaterrestris* was subsequently synonymized with *N.brevipes* by Yaginuma himself (1972: p. 619–621) believing that the differences in the shape of male palp and epigyne were part of the intraspecific variability of the species. Both the morphological and molecular results illustrated in our study clearly show that *N.terrestris* and *N.brevipes* are in fact two distinct species. Based on these conclusions the resurrection of *N.terrestris* as a valid species is herein proposed. Accordingly, we describe and illustrate for the first time the male of *N.terrestris* based on specimens collected from the type locality of the species. Due to the synonymization of *N.terrestris* with *N.brevipes* the published records of these two species are currently mixed. In this study we considered only the records that we could directly confirm on the basis of published drawings or checked samples. Based on our material all records from central and eastern Honshu up to Hokkaido refer to *N.terrestris*, while *N.brevipes* can be found in the islands of Honshu, Kyushu, and part of the Kansai area. (Fig. 16A). Due to the lack of specimens from western Kansai and Chugoku areas we cannot confirm the presence of *N.terrestris* in western Honshu although it seems plausible. Specimens of *N.brevipes* from Kuril Is. (Marusik and Crawford 2006) were checked by us and confirmed as *N.terrestris*. The illustrated specimens of *N.brevipes* from Korea (e.g., Kim and Lee 2018, fig. 11a–c) show close similarities with the palp and epigyne of *N.terrestris* rather than *N.brevipes* and possibly refer to the this or another closely related species (cf. Figs 5A–G, 7A–D vs Figs 6A–G, 7E–H vs Kim and Lee 2018: fig. 11A–C). The presence of *N.terrestris* in Korea needs to be properly verified directly examining the specimens.

### ﻿ Nesticella silvicola sp. nov.

Taxon classificationAnimaliaAraneaeNesticidae

DDD0D759-91DF-5D8A-B0EA-5CF61A6D8B91

https://zoobank.org/F33130C7-220A-48BC-9CA2-4E3D7715867F

Figs 8A–G , 10A–D , 16B (Japanese name: Yamako-horahimegumo ヤマコホラヒメグモ)

#### Material examined.

♂ ***Holotype*: Japan: Kagoshima Pref., Yakushima Is.**: Koseda, Nagamine, 29.Mar.2023, S. Konishi leg. (NSMT-Ar 25254).

***Paratypes*: Japan: Kagoshima Pref., Yakushima Is.**: 1♀, Koseda, 190 m, broadleaf forest litter on a gentle slope, 30.38286°N, 130.62455°E, 24.Sep.2021, F. Ballarin leg. (RMUF); 2♀, Isso, 130 m, broadleaf forest litter bordering a sugi plantation near a river, 30.43615°N, 130.48129°E, 27.Sep.2021, F. Ballarin leg. (NSMT-Ar 25255); 1♀, Miyanoura, 15.July.1990, A. Tanikawa leg. (FBPC); 1♀, Miyanoura, 341 m, humid broadleaf forest litter in a humid valley near a creek, 30.39696°N, 130.55584°E, 27.Sep.2021, F. Ballarin leg. (FBPC); 1♀, Anbo, 224 m, broadleaf forest litter, 30.28375°N, 130.61619°E, 27.Sep.2021, F. Ballarin leg. (FBPC); 1♀ Jhonji-dake, 18.Feb.2023, S. Konishi leg. (NSMT-Ar 25256)

#### Etymology.

The specific name is derived from the Latin adjective *silvicolus* (= inhabiting woods, sylvan). It refers to the habitat of the species, living in the forests of Yakushima Is.

#### Diagnosis.

This species is closely related to *N.brevipes* and *N.terrestris*. Male of *N.silvicola* sp. nov. can be distinguished from male of *N.terrestris* by the stockier distal process of paracymbium (Di), the sharper radical apophysis (Ra), and by the wider ventral process II of paracymbium (Ve-II), (vs sharper Di, rounder Ra, and thinner Ve-II in *N.terrestris*). (Figs 8A–D, 10A–C cf. Figs 6A–D, 7E–G). In addition, the origin of the embolus (E) from the radix is located in a different position in the two species (4:30 o’clock in *N.silvicola* vs 6:00 o’clock in *N.terrestris*) (Figs 8A, 10A cf. Figs 6A, 7). Male of the new species can be easily separated from male of *N.brevipes* by the single distal process of paracymbium (Di), a wider radical apophysis (Ra), and a thicker median process of conductor (Cm) (vs two Di, a slimmer Ra, and a thinner Cm in *N.brevipes*). (Figs 8A–D, 10A–C cf. Figs 5A–D, 7A–C).

Female of *N.silvicola* sp. nov. can be distinguished from female of the other Japanese congeners of the *N.brevipes* group, by the general shape of genitalia, having a short scapus (Sc) with a flat distal margin, more squared lateral margins and strongly bent internal ducts (vs a longer and lobated Sc with straight ducts in *N.brevipes*, a more lobated scapus with rounder distal margins and straight internal ducts in *N.terrestris*, or a narrower and more trapezoidal scapus in *N.insulana* sp. nov. (Figs 9E–G, 10F cf. Figs 5E–G, 6E–G, 7D, H, 8E–G, 10D). The smaller size further allows a quick separation of *N.silvicola* sp. nov. from *N.terrestris* (females 1.84–1.94 vs 2.31–3.30).

#### Description of male

**(holotype).** Habitus as in Fig. 8H. Total length: 1.91. Prosoma 0.97 long, 0.86 wide. Carapace rounded, yellowish with slightly darker margins and central area. Cervical groove and fovea distinct. Eyes well developed. Eyes measurements: AME = 0.04, ALE = 0.08, PME = 0.08, PLE = 0.08, AME–ALE = 0.03, ALE–PLE = 0.00. Chelicerae, labium, maxillae, and sternum of same color as carapace. Legs yellowish with slightly darker annulation on distal femur, tibia, and metatarsus. Legs measurements: I 5.63 (1.60, 0.43, 1.48, 1.48, 0.64), II 4.28 (1.25, 0.38, 1.05, 1.06, 0.54), III 3.91 (1.00, 0.31, 0.82, 0.81, 0.47), IV 4.58 (1.44, 0.41, 1.16, 1.13, 0.44). Opisthosoma greyish with couples of slightly darker marks on anterior and dorsal side gradually merging to each other toward the posterior side.

Palp as in Figs 8A–D, 10A–C. Cymbium relatively short, covered with sparse setae, some thicker setae on distal-prolateral margin (Fig. 8D). Paracymbium with a single distal process (Di), two ventral processes (Ve-I–II), and a dorsal apophysis (Do): distal process (Di) stumpy, headed ventrally and retrolaterally, hook-like when observed laterally; ventral process I (Ve-I) sharp and slim, headed internally; ventral process II (Ve-II) lobated, headed internally; dorsal apophysis (Do) lobated, wide and flat (Figs 8A–D, 10A–C).Embolus (E) long and filiform, origin of embolus positioned at ~ 4:30 o’clock on radix (Rx). Radical apophysis (Ra) strongly sclerotized, wide and stumpy, triangular, ending with a rounded tip. Conductor with 3 distinct processes (Cp, Cr, Cm) and a half-transparent distal lobe (Cl). Prolateral process of conductor (Cp) flat, ribbon-like and headed counterclockwise, wrapped around embolus. Retrolateral process of conductor (Cr) wide and thick, curved inside. Median process of conductor (Cm) thick and strongly sclerotized, spine-like, ending blunt, with a ribbon-like lobe wrapped around its prolateral side. (Figs 8A–C, 10A, B).

#### Description of female

**(one of the paratypes).** Habitus as in Fig. 8I. Total length: 1.87. Prosoma 0.88 long, 0.78 wide. Cephalic area as in Fig. 8J. Eyes measurements: AME = 0.04, ALE = 0.08, PME = 0.08, PLE = 0.08, AME–ALE = 0.03, ALE–PLE = 0.00. Coloration and other details as in male. Legs measurements: I 4.65 (1.33, 0.40, 1.21, 1.10, 0.61), II 3.68 (1.10, 0.37, 0.86, 0.82, 0.53), III 2.09 (0.89, 0.30, 0.62, 0.63, 0.46), IV 3.96 (1.22, 0.33, 1.02, 0.88, 0.53).

Epigyne and vulva as in Figs 8E–G, 10D. Scapus (Sc) very short and wide, rectangular, ~ 2.5× wider than long, ending with a flat posterior margin slightly concave in the center (Figs 8E, F, 10D). Copulatory opening (Co) at the inner-lateral sides of scapus. Internal ducts slightly visible through the transparent tegument, shaped as 2 angled brackets pointing towards each other. Copulatory ducts (Cd), short and thick, bent in the central trait, proximal part of ducts heading slightly internally, distal part heading laterally before reaching spermathecae. Insemination ducts (Id) thin, coiled around copulatory ducts. Spermathecae (S) small and rounded, separated from each other by ~ 1.5× their diameter (Fig. 8G).

#### Size variation.

(based on 5 females). Total length: 1.84–1.94, prosoma length: 0.86–0.99, prosoma width: 0.78–0.84.

#### Distribution.

Known only from Yakushima Is. in western Japan (Fig. 16B).

#### Habitat and ecology.

*Nesticellasilvicola* sp. nov. inhabits the shadowed and humid broadleaf and mixed forests covering the mountain slopes in Yakushima Is. This species builds simple scaffold webs in the empty spaces and recesses between the leaf litter and under rotten logs.

### ﻿ Nesticella insulana sp. nov.

Taxon classificationAnimaliaAraneaeNesticidae

AE62E405-4D24-52F5-898E-4D62E5832E7E

https://zoobank.org/9961274C-5928-4C53-9E5D-094C11C4C839

Figs 9A–J , 10E–H , 16B (Japanese name: donan-horahimegumo ドナンホラヒメグモ)

#### Material examined.

♂ ***Holotype*: Japan: Okinawa Pref., Yonaguni-jima Is.**: Mantabaru Forest Park (満田原森林公園), 54 m, humid forest litter in a narrow valley, 24.45652°N, 122.97692°E, 3.Mar.2021, K. Eguchi leg. (NSMT-Ar 25257).

***Paratypes***: 4♀, same locality and date as the holotype, K. Eguchi leg. (NSMT-Ar 25258); 5♀, same locality and date, 112 m, 24.45678°N, 122.97675°E, F. Ballarin leg. (FBPC).

#### Other material examined.

1♂ subad., same locality and date as the holotype, 115 m, humid forest litter, 24.45660°N, 122.97584°E, F. Ballarin leg. (FBPC); 1♀ subad., 54 m, humid forest litter in a small valley near route 216, 24.44740°N, 122.96827°E, 04.Mar.2021, F. Ballarin leg. (FBPC).

#### Etymology.

The specific name is derived from the Latin adjective *insulanus* (= islander). It refers to the distribution of the species, limited to the island of Yonaguni-jima.

#### Diagnosis.

The new species is closely related to *Nesticellaodonta* (Chen, 1984) from Zhajinag Province, China. Male of the new species can be distinguished from male of *N.odonta* by the wider distal process I of paracymbium (Di-I), the smaller and blunter distal process II (Di-II), and by the more curved ventral process II (vs thinner and sharper Di-I, wider Di-II, and straighter Ve-II in *N.odonta*) (Figs 9A, B, D, 10E–G cf. Lin et al. 2016: figs 24A, B, D). Female of *N.insulana* sp. nov. can be separated from female of *N.odonta* by the narrower, longer, and more arrow-like scapus (Sc) of the epigyne (vs wider, more trapezoidal, and shorter scapus in *N.odonta*) (Figs 9E–G, 10H cf. Lin et al. 2016: fig. 25E–G). The general shape of genitalia in both male and female allows to easily discern *N.insulana* sp. nov. from all the other Japanese congeners of the *N.brevipes* group.

#### Description of male

**(holotype).** Habitus as in Fig. 9H. Total length 1.81. Prosoma 0.91 long, 0.83 wide. Carapace rounded, yellowish with darker areas around borders and central area. Cervical groove and fovea distinct. Eyes well developed. Eyes measurements: AME = 0.05, ALE = 0.08, PME = 0.08, PLE = 0.08, AME–ALE = 0.04, ALE–PLE = 0.01. Chelicerae, labium, maxillae, and sternum of same color as carapace. Legs yellowish often with dark annulations on femur, and tibia. Legs measurements as follows: I 5.98 (1.65, 0.43, 1.58, 1.59, 0.73), II 4.35 (1.25, 0.31, 1.13, 1.06, 0.60), III 3.33 (0.98, 0.36, 0.70, 0.76, 0.53), IV 4.67 (1.43, 0.33, 1.19, 1.04, 0.68). Opisthosoma greyish with large dark marks on dorsal and lateral sides.

Male palp as in Figs 9A–D, 10E–G. Cymbium relatively short, 4–6 thicker setae on distal-prolateral margin (Fig. 9D). Paracymbium with 2 distal processes (Di-I, II) and 2 flat, lobated ventral processes (Ve-I–II): distal process I (Di-I) wide and lobated, hook-like when observed laterally, with tip headed ventral-retrolaterally; distal process II (Di-II) short and blunt, headed frontally; ventral process I (Ve-I) shorter, headed anteriorly; ventral process II (Ve-II) long and curved, headed antero-retrolaterally (Figs 9A–D, 10E–I Embolus (E) long and filiform, origin of embolus positioned at ~ 4:00 o’clock on radix (Rx). Radical apophysis (Ra) strongly sclerotized, triangular, and sharp, ending with a pointed tip. Conductor with 3 distinct processes (Cp, Cr, Cm) and a half-transparent distal lobe (Cl). Prolateral process of conductor (Cp) flat, ribbon-like, headed counter-clockwise, wrapped around embolus. Retrolateral process of conductor (Cr) wide and thick, curved inside, bearing a small sclerotized apophysis (Ca). Median process of conductor (Cm) strongly sclerotized, thin, spine-like ribbon-like, with a ribbon-like lobe wrapped around its prolateral side. (Figs 9A–C, 10E, F).

#### Description of female

**(one of the paratypes).** Habitus as in Fig. 9I. Total length 2.05. Prosoma 0.91 long, 0.84 wide. Cephalic area as in Fig. 9J. Carapace piriform. Eyes measurements: AME = 0.05, ALE = 0.07, PME = 0.08, PLE = 0.08, AME–ALE = 0.04, ALE–PLE = 0.01. Coloration and other details as in male. Legs measurements as follows: I 5.45 (1.61, 0.45, 1.41, 1.33, 0.65), II 3.98 (1.19, 0.41, 0.93, 0.89, 0.56), III 3.04 (0.88, 0.34, 0.67, 0.64, 0.51), IV 4.29 (1.39, 0.40, 1.09, 0.92, 0.49).

Epigyne and vulva as in Figs 9E–G, 10H. Scapus (Sc) short and stumpy, arrow-like, ~ 2× wider than long, with sloped borders and narrower, slightly rounded tip (Figs 9E, F, 10H). Copulatory opening (Co) at the inner-lateral sides of scapus. Internal ducts slightly visible through the transparent tegument, shaped as curly bracket. Copulatory ducts (Cd) thick, strongly bent in middle trait, curving first laterally and then anteriorly before reaching spermathecae. Insemination ducts thin, coiled around copulatory ducts. Spermathecae (S) small and rounded, separated from each other by ~ 1.5× their diameter (Fig. 9G).

#### Size variation.

Female (based on 5 specimens): total length 1.90–2.57, prosoma length: 0.82–1.05, prosoma width: 0.78–0.93.

#### Distribution.

Endemic to Yonaguni-jima Is., western Ryukyus. Known only from the type locality (Fig. 16B).

#### Habitat and ecology.

*Nesticellainsulana* sp. nov. inhabits the shadowed and humid forests covering the hills of the central-western area of Yonaguni-jima Island. This species builds simple scaffold webs in the empty spaces among the leaf litter accumulated in narrow valleys, under rotten wood and on vegetated cliffs. Despite several attempted surveys, this species was not collected in other parts of the island.

##### ﻿*Nesticellaquelpartensis* group

### ﻿ Nesticella occulta sp. nov.

Taxon classificationAnimaliaAraneaeNesticidae

13C2D1DC-D3E0-540C-B9D3-3C3AB2B08F6B

https://zoobank.org/3DF252B6-7D9D-47B4-82A7-95E6F223190A

Figs 11A–E ; 13F , 15D , 16B (Japanese name: kakure-horahimegumo カクレホラヒメグモ)

#### Material examined.

♀ ***Holotype*: Japan: Okinawa Pref.: Ishigaki-jima Is.**: Kabira, Kabirano-ana cave (川平の穴), long and superficial cave with many rocks, in the dark zone of the cave, 24.47384°N, 124.13416°E, 20.Sep.2022, F. Ballarin leg. (NSMT-Ar 25259). ***Paratypes***: 1♀, same locality as the holotype, 1.Apr.2019, K. Uchida leg. (NSMT-Ar 25260); 1♀, same locality, 2.Jan.2022, K. Uchida leg. (RMUF); 2♀, same locality and date as the holotype, F. Ballarin leg. (FBPC).

#### Other material examined.

1♂subad., same locality as the holotype, 16.Oct.2020; 1♂subad., same locality, 2.Jan.2022; 1♂subad., same locality, 28.Mar.2022, all K. Uchida leg. (RMUF).

#### Etymology.

The specific name derives from the Latin adjective *occultus* (= hidden, secret). It refers to the troglobitic lifestyle and rarity of the species, hidden in the deep recess of a single cave in Ishigaki-jima Is.

#### Diagnosis.

This species closely related to *N.kaohsiungensis* Lin, Ballarin & Li, 2016 from Taiwan. The new species can be easily distinguished from *N.kaohsiungensis* by the strongly reduced eyes and the lack of pigmentation and pattern on the opisthosoma (vs eyes well-developed, clear pattern on the opisthosoma and pigmentation present in *N.kaohsiungensis*) (Fig. 11A, B cf. Lin et al. 2016: fig. 69A, C). In addition, the female of this species can be distinguished from the female of *N.kaohsiungensis* by the different shape of scapus, lacking a lobated tip (vs ending with a lobated tip in the latter species) (Figs 11C, D, 13F cf. Lin et al. 2016: fig. 69E, F).

#### Description.

**Female (holotype).** Habitus as in Fig. 11A, 15D. Total length 2.48. Prosoma 1.08 long, 0.93 wide. Carapace piriform, uniformly pale yellowish. Cephalic area as in Fig. 11B. Eyes completely degenerated. Cervical groove and fovea indistinct. Chelicerae uniformly brownish. Labium, maxillae, and sternum pale yellowish as carapace. Legs uniformly pale yellowish. Legs measurements: I 6.36 (2.18, 0.47, 1.48, 1.46, 0.77), II 6.13 (1.97, 0.45, 1.60, 1.42, 0.69), III 4.36 (1.43, 0.38, 0.95, 0.90, 0.70), IV 6.02 (1.94, 0.46, 1.54, 1.32, 0.76). Opisthosoma uniformly greyish, covered by long, sparse hairs.

Epigyne and vulva as in Figs 11C–E, 13F. Scapus (Sc) long and protruding, tongue-like, ~ 2× longer than wide, ending with a rounded tip (Figs 11C, D, 13F). Copulatory opening (Co) at the inner-lateral sides of scapus. Internal ducts slightly visible through the transparent tegument, V shaped. Copulatory ducts (Cd) short and straight, strongly diverging from each other (Figs 11C–E, 13F) distal trait coiled, reaching spermathecae with 1 coil. Insemination ducts (Id) thin, coiled around the copulatory ducts. Spermathecae (S) small and rounded, separated from each other by ~ 2.5× their diameter (Fig. 11E).

**Male.** Unknown.

#### Size variation.

(based on 4 females) Total length: 1.75–2.48, prosoma length: 1.06–1.10, width: 0.92–0.94 wide.

#### Distribution.

Ishigaki-jima Island. Known only from the type locality (Fig. 16B).

#### Habitat and ecology.

*Nesticellaocculta* sp. nov. lives in the dark zone of the type locality cave characterized by high and stable temperature and humidity (e.g., temp: 25.8 °C, hum: 94.4%) (Fig. 15H). It builds small and simple scaffold-webs among the crevices and empty spaces between the numerous rocks covering the floor of the cave and on the lower part of the cave walls. These spiders appear scattered inside the cave and rather infrequently, suggesting a relatively small population. The compete eyes degeneration, the lack of pigmentation and the presence of the species only in the deepest section of the cave suggest that *N.occulta* sp. nov. is a true troglobiont. These characteristics as well as the rarity of the species, inhabiting a single cave, make *N.occulta* sp. nov. a good target for species conservation.

##### ﻿*Nesticellaokinawaensis* group

### ﻿ Nesticella okinawaensis

Taxon classificationAnimaliaAraneaeNesticidae

(Yaginuma 1979)

A74275A7-B1BF-5C15-98CA-279FCA285E09

Figs 12A–J , 13A–D , 15E , 16B (Japanese name: okinawa-horahimegumo オキナワホラヒメグモ)

Nesticus okinawaensis Yaginuma 1979: 275, pl. 6, figs 6–8 (♂♀): Yaginuma 1986: 55, fig. 31.7 (♂♀).
Howaia okinawaensis Lehtinen and Saaristo 1980: 59 (♂♀, transferred from Nesticus).
Nesticella okinawaensis Kamura and Irie 2009: 353, figs 111, 112 (♂♀).

#### Type locality.

Japan, Okinawa honto Is., Kakinohana,Tamagusuku-son, Yaaji-gama Cave.

#### Material examined.

**Japan: Kagoshima Pref.: Amami-Ōshima Is.**: 1♂, 1♀, Amami-shi, Naze, forest litter, 15.Mar.2020, R. Serita leg. (FBPC); **Okinoerabu-jima Is.**: 1♀, Murauchi Shindō cave (村内新洞), 4.May.2004, H. Tamura leg. (MNHAH); 1♀, China-cho, Ginsuido cave (銀水洞), 27.Apr.2004, H. Tamura leg. (MNHAH); **Okinawa Pref.: Okinawa-honto Is.**: 2♀, Kunigami-gun, Motobu-cho, Izumi, 216 m, forest litter along the road, 26.63968°N, 127.93916°E, 17.Nov.2020; 1♀, Kunigami-son, Yona, Yambaru Park, 185 m, humid forest litter, 26.74755°N, 128.22347°E, 25.Feb.2021; 1♂, 10♀, same locality, 132 m, 26.75168°N, 128.22227°E, 28.Feb.2021; 2♀, same locality, 206 m, 26.74536°N, 128.22545°E, 28.Feb.2021; 2♀, same locality, 55 m, 26.75803°N, 128.22167°E, 01.Mar.2021, all F. Ballarin leg. (FBPC); 1♂, 1♀, Ogimi-son, Okuni-rindo Pass, 7.Mar.2020, Y. Suzuki leg. (YSPC); **Kume-jima Is.**: 2♀, Shimajiri-gun, Maja, 95 m, litter in a broadleaf forest, 26.34819°N, 126.80254°E, 18.May.2022, F. Ballarin leg. (FBPC); 1♀, Uegusuku, 263 m, litter in a broadleaf forest, 26.37577°N, 126.76990°E, 18.May.2022, F. Ballarin leg. (FBPC).

#### Diagnosis.

Male of *Nesticellaokinawaensis* can be distinguished from male of other Japanese congeners by the short and stocky beak-like median process of conductor (Cm) with a long ventral process (vs a longer, smaller, or thinner Cm, with a smaller or lacking ventral process in other species), by the presence of a long, lobated distal apophysis on the retrolateral process of conductor (Ca) (vs small Ca in *N.silvicola* sp. nov. or lacking in other species), and by the shape of paracymbium having an elongated distal process I (Di-I) and a lobated and complex distal process II (Di-II) (vs a shorter Di-I, and a missing, simpler, smaller or thinner Di-II in other species) (Figs 12A–D, 13A–C cf. Figs 1A–D, 2A–D, 4A–C, E–G, 5A–D, 6A–D, 7A–C, E–G, 8A–D, 9A–D, 10A–C, E–G). Female of *N.okinawaensis* is easily distinguished from female of other Japanese species by the shape of the internal copulatory ducts (Cd), thin and convoluted (vs thicker and less convoluted Cd in other species) (Figs 12E–G, 13D cf. Figs 1E–G, 2E–G, 3D, E, 4D, H, 5E–G, 6E–G, 7D, H, 8E–G, 9E–G, 10D, H, 11C–E, 13E, F).

#### Redescription of male

**(from Yambaru Park, Okinawa-honto).** Habitus as in Fig. 12H. Total length 1.89. Prosoma 1.02 long, 0.90 wide. Carapace rounded, yellowish with dark cephalic area, median stripe, and margins. Cervical groove and fovea distinct. Eyes well developed. Eyes measurements: AME = 0.04, ALE = 0.09, PME = 0.09, PLE = 0.09, AME–ALE = 0.03, ALE–PLE = 0.00. Chelicerae, labium, maxillae, and sternum of the same yellowish color as carapace. Legs yellowish with dark annulation on femur, patella, tibia, metatarsus, and tarsus. Legs measurement: I 5.46 (1.51, 0.40, 1.41, 1.41, 0.53), II 4.68 (1.41, 0.38, 1.17, 1.16, 0.56), III 3.43 (1.07, 0.33, 0.77, 0.83, 0.43), IV 5.15 (1.57, 0.41, 1.39, 1.21, 0.57). Opisthosoma dark grey with whitish dorsal mark on dorsal-anterior side.

Male palp as in Figs 12A–D, 13A–C. Cymbium elongated, five or six robust setae on the distal and distal-prolateral margin (Fig. 12D). Paracymbium with 2 distal processes (Di-I–II) and 1 ventral process (Va). Distal process I (Di-I) long, laterally flattened, slightly bent internally, ending with a small lobated tip; distal process II (Di-II) wide, proximal part laterally flattened, headed frontally, distal part lobated, headed internally bearing 2 small spurs. Ventral process of paracymbium (Va) wide, flat, triangularly shaped. (Figs 12A, B, D, 13A, C). Embolus (E) long and filiform, origin of embolus positioned at ~ 4:30 o’clock on radix (Rx). Radical apophysis (Ra) strongly sclerotized, rectangularly shaped, flat, and stocky with granulated surface. Conductor with 3 distinct processes (Cp, Cr, Cm) and a half-transparent distal lobe (Cl). Prolateral process (Cp) long and flat, ribbon-like, headed counterclockwise, wrapped around embolus. Retrolateral process (Cr) wide and thick, curved internally, distally bearing a long, lobated apophysis (Ca) and a flat, triangular outgrowth in the central part. Median process of conductor (Cm) beak-like, short and stocky, strongly sclerotized, with a long, strongly sclerotized ventral process (Figs 12A–C, 13A, B).

#### Redescription of female

**(from Yambaru Park, Okinawa-honto).** Habitus as in Figs 12I, 15E. Total length 1.84. Prosoma 0.87 long, 0.72 wide. Carapace piriform. Cephalic area as in Fig. 12J. Eyes well-developed. Eyes measurements: AME = 0.03, ALE = 0.08, PME = 0.08, PLE = 0.08, AME–ALE = 0.03, ALE–PLE = 0.00. Legs measurements: I 6.13 (1.51, 0.41, 1.36, 1.28, 0.57), II 3.66 (1.09, 0.36, 0.85, 0.83, 0.53), III 2.58 (0.70, 0.31, 0.56, 0.55, 0.46), IV 4.26 (1.33, 0.40, 1.04, 0.98, 0.51). Coloration and other details as in male.

Epigyne and vulva as in Figs 12E–G, 13D. Scapus (Sc) short, rectangular, elongated laterally, ~ 2× wider than long, bearing a flat posterior margin (Figs 12E, F, 13D). Copulatory opening (Co) at the inner-lateral sides of scapus. Internal ducts slightly visible through the transparent tegument, shaped as 2 inverted round brackets. Copulatory ducts (Cd), thin, coiled, reaching the spermathecae after 2 coils. Insemination ducts (Id) thin. Spermathecae (S) small and rounded, separated from each other by ~ 2.5× their diameter (Fig. 12G).

#### Size variation.

Male (based on 3 males) Total length: 1.85–1.89, prosoma length: 1.01–1.02, width: 0.88–0.90. Female (based on 8 females) Total length: 1.68–2.07, prosoma length: 0.88–0.92, width: 0.79–0.81.

#### Distribution.

*Nesticellaokinawaensis* is distributed in the islands forming the Central Ryukyu arc (Fig. 16B). The new records for Amami-Ōshima and Okinoerabu-jima islands herein reported extend the known distribution of this species ~ 200 km to the North-East. Currently, *N.okinawaensis* is recorded from the islands of Amami-Ōshima, Okinoerabu-jima, Okinawa-honto, and Kume-jima (Yaginuma 1979; Tanikawa and Sasaki 1999; and this work) but its presence in other minor islands of the Central Ryukyu is also probable.

#### Habitat and ecology.

*Nesticellaokinawaensis* dwells in humid and shadowed habitats such as caves, narrow valleys, and vegetated cliffs. This species builds simple scaffold webs in empty spaces in the leaf litter or under logs and superficial rocks, as well as in recesses of the walls and floor of caves, usually in the twilight zone.

#### Remarks on intraspecific variation.

The general coloration and the dorsal pattern of the opisthosoma appear rather variable in *N.okinawaensis*, changing among populations living in different areas or different islands or even single individuals. Populations living in the northern area of Okinawa-honto Is. often show a darker pattern, with less clear marks often reduced to a single whitish spot on the dorsal-anterior side (Figs 12I, 15E); populations from Kume-jima Is. often have a whitish continuous stripe along the median line of the opisthosoma, while populations from Amami-Ōshima Is. usually have a general lighter pattern with numerous larger light marks partially fused to each other (Fig. 12H). The shape of scapus also shows minor variability among specimens from different islands with the samples from Kume-jima Is. and Okinoerabu-jima Is. generally having a slightly narrower scapus. A high degree of genetic variation is also observed between population distributed in different islands (e.g., Amami Is.-Kumejima Is. = 7.3–7.8%; Amami Is.-Okinawa Is. = 7.5%; Kumejima Is.-Okinawa Is. = 5.6–6.7%).

#### Remarks on systematic.

*Nesticellaokinawaensis* was provisionally included in the *N.brevipes* group by Lin et al. (2016) on the basis of published drawings only. Our molecular analysis, and a detailed morphological comparison of this species with the type material of *N.brevipes*, suggest that *N.okinawaensis* is in fact far related to the *N.brevipes* group and it belongs to a distinct clade. In addition, this species seems to have a basal position within the genus *Nesticella* (Fig. 14). Nevertheless, the phylogeny reconstructed in this work is based on a single gene fragment and includes only a part of the known *Nesticella* species. Preliminary analysis including more species (e.g., species of the *N.phami* group) seem to confirm the monophyly of *N.okinawaensis* although the support of the main nodes decreases substantially. Wider and more detailed molecular and morphological studies including a larger number of species and gene fragments are necessary to confirm the phylogenetic position of *N.okinawaensis* within *Nesticella*.

## Supplementary Material

XML Treatment for Howaia

XML Treatment for Howaia mogera

XML Treatment for Howaia alba

XML Treatment for Howaia subterranea

XML Treatment for Nesticella

XML Treatment for Nesticella brevipes

XML Treatment for Nesticella terrestris

XML Treatment for Nesticella silvicola

XML Treatment for Nesticella insulana

XML Treatment for Nesticella occulta

XML Treatment for Nesticella okinawaensis

## Footnotes

For the complete list of references see the World Spider Catalog 2023.

## ﻿Additional information

### Author ORCIDs

Francesco Ballarin https://orcid.org/0000-0003-1417-2519

Katsuyuki Eguchi https://orcid.org/0000-0002-1054-1295

### Data availability

All of the data that support the findings of this study are available in the main text.
